# On-demand anchoring of wireless soft miniature robots on soft surfaces

**DOI:** 10.1073/pnas.2207767119

**Published:** 2022-08-15

**Authors:** Ren Hao Soon, Ziyu Ren, Wenqi Hu, Ugur Bozuyuk, Erdost Yildiz, Meng Li, Metin Sitti

**Affiliations:** ^a^Physical Intelligence Department, Max Planck Institute for Intelligent Systems, 70569 Stuttgart, Germany;; ^b^Institute for Biomedical Engineering, ETH Zürich, 8092 Zürich, Switzerland;; ^c^School of Medicine, Koç University, 34450 Istanbul, Turkey;; ^d^College of Engineering, Koç University, 34450 Istanbul, Turkey

**Keywords:** Wireless medical robots, soft robots, miniature robots, surface anchoring, medical devices

## Abstract

Untethered soft miniature robots capable of accessing hard-to-reach regions can enable new, disruptive, and minimally invasive medical procedures. However, once the control input is removed, these robots easily move from their target location because of the dynamic motion of body tissues or fluids, thereby restricting their use in many long-term medical applications. To overcome this, we propose a wireless spring-preloaded barbed needle release mechanism, which can provide up to 1.6 N of force to drive a barbed needle into soft tissues to allow robust on-demand anchoring on three-dimensional (3D) surfaces. The mechanism is wirelessly triggered using radio-frequency remote heating and can be easily integrated into existing untethered soft robotic platforms without sacrificing their mobility. Design guidelines aimed at maximizing anchoring over the range of the most biological tissues (kPa range) and extending the operating depth of the device inside the body (up to 75%) are also presented. Enabled by these advances, we achieve robust anchoring on a variety of ex vivo tissues and demonstrate the usage of such a device when integrated with existing soft robotic platforms and medical imaging. Moreover, by simply changing the needle, we demonstrate additional functionalities such as controlled detachment and subsurface drug delivery into 3D cancer spheroids. Given these capabilities, our proposed mechanism could enable the development of a new class of biomedical-related functionalities, such as local drug delivery, disease monitoring, and hyperthermia for future untethered soft medical robots.

## Significance

Anchoring soft millirobots on surfaces, such as biological tissues, is essential to perform long-duration medical functions robustly on a target position. For robust anchoring, we propose a wireless mechanism that can be precisely controlled by remote heating to achieve on-demand needle release and mechanical interlocking. Such a mechanism can be easily integrated on existing untethered soft robots, allowing them to anchor robustly to soft surfaces while retaining their locomotion capabilities. Furthermore, we demonstrate advanced functionalities of such robots, such as controlled surface detachment and subsurface drug delivery into three-dimensional cancer spheroids. Given these capabilities, the proposed mechanism can serve as a platform for the development of soft robots with a new suite of biomedical capabilities.

Wireless miniature mobile robots capable of navigating inside hard-to-reach sites of our body have great potential in minimally invasive medical applications (1–3). In particular, magnetically actuated and controlled wireless miniature soft robots are highly promising, as external magnetic fields are wireless, dexterous, and precise and can safely penetrate deep inside the human body (4–6). Recent advancements in this field have enabled these robots to navigate to a precise target position on land (7, 8) or in a fluid-filled confined environment (9, 10). Moreover, scenarios involving local cargo delivery, such as hydrogel structures (11), drugs (12–15), genes (16), imaging contrast agents, and stem cells (17), have been demonstrated, highlighting the potential use of these robots in biomedical contexts. However, the requirement of a long-lasting magnetic field input could severely limit the use of miniature magnetic medical robots in certain clinical applications, especially those requiring extended operation durations inside the body. Once the magnetic field input is removed, the robots would lose all actuation capabilities, and they would easily move away from their target position due to the dynamic motion of the body tissues and fluids and other disturbances.

Although recent advances in the development of milliscale and microscale functional materials, manufacturing, and assembly techniques (6, 18–20) have enabled integrated anchoring mechanisms for wireless medical devices, they are mostly limited to confined tubular structures (20). Moreover, existing strategies for surface attachment that exploit mechanical interlocking (21–23), van der Waals force (24, 25), capillary force (26), suction force (27), or a combination of these forces (28, 29) do not perform well on biological tissues for various reasons. First, internal tissue surfaces are highly wet, textured, rough, and covered with various biological complex fluids, such as a constantly regenerating mucus layer (30, 31). These properties reduce the surface contact area and hence the maximum adhesion. Even if strong adhesion is achieved in special conditions, it could be only temporary since the mucus layer would be replaced gradually over time (32). Second, although some of these methods can attach well to biological surfaces, they require a high preload and have limited attachment testing on dynamic loads (33, 34). Such a requirement makes them incompatible with miniature soft robots with limited force outputs (*SI Appendix*, *Theoretical Force Provided by a Magnetic Soft Robot*). With these issues, for reliable one-time long-term anchoring, the mechanism should directly engage with the underlying tissue layer (*SI Appendix*, Table S1). We rule out the chemical methods because there are more issues to consider as compared to physical attachment methods (35). For instance, mucoadhesion, a common chemical method of attachment, is heavily dependent on both internal (e.g., pH, concentration of drugs loaded, hydrophilicity, and molecular weight) and external factors (e.g., specific formulations that have to be designed for each surface and swelling because of working environment) (36–38). These factors are hard to predict and monitor in real applications, and variance in any of these factors may prevent robust anchoring.

**Fig. 1.**
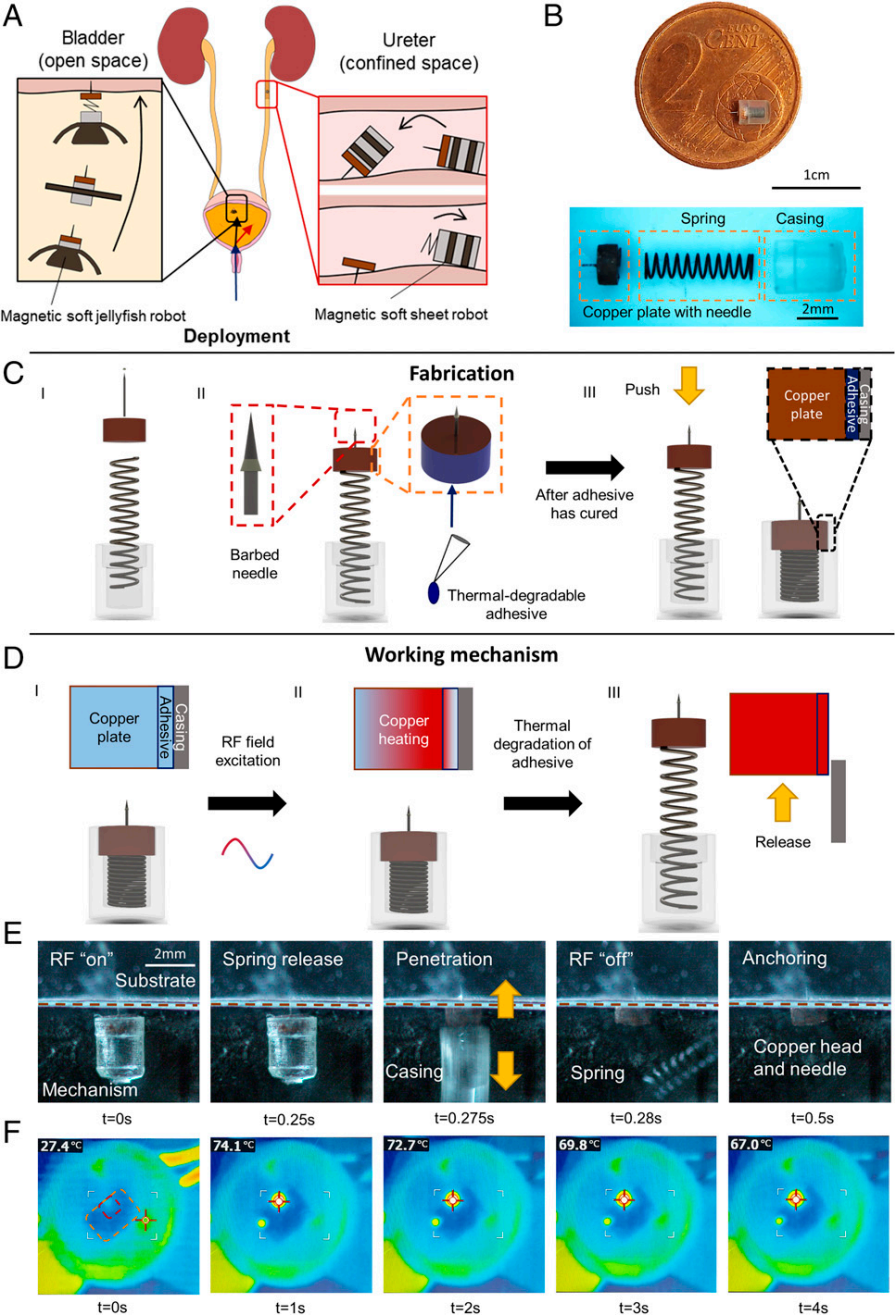
Proposed RF-triggered spring-loaded surface-anchoring mechanism. (*A*) Conceptual illustration of the anchoring mechanism integrated with existing soft magnetic robots to achieve anchoring in open and enclosed environments. (*B*) Fabricated prototype. (*C*) Fabrication procedure. (*C*, *I*) The components (barb, needle, copper plate, spring, and casing) are brought together. (*C*, *II*) Cyanoacrylate adhesive is applied to the sides of the copper plate, and the barb is mounted on the needle and secured with adhesive. (*C*, *III*) The copper plate is then pushed into the casing after the adhesive has cured to create an interference fit. (*D*) Actuation sequence of the mechanism. (*Insets*) The temperature changes inside the copper plate as the mechanism is triggered. Red and blue represent higher and lower temperatures, respectively. (*D, I*) The mechanism is positioned. (*D, II*) Exposure to RF field heats up the copper plate. (*D, III*) Device is triggered (*E*) High-speed video image snapshots of the firing process in air. The brown line indicates the boundary between the substrate and the mechanism. The images are blurred primarily because some parts are moving out of plane, and the firing process takes place too quickly for the focus to be adjusted automatically or manually. The arrows indicate the direction of movement of the various parts as the mechanism is fired. (*F*) Infrared camera images of the firing process in air. In the first infrared image, the area bordered in orange indicates the boundaries of the substrate, and the area bordered in red indicates the approximate location of the mechanism. Cross from t = 1 s to t = 4 s indicates the location of the hottest region, which corresponds to where the copper plate is located.

Here we propose a mechanism that can be precisely controlled by an external radio frequency (RF) field-based heating to achieve on-demand needle release (Fig. 1*A*). In addition, the small size of our proposed mechanism (2.7 mm diameter and 3.2 mm compressed length) allows it to not only access hard-to-reach regions inside the human body but also be readily integrated on soft millirobots (Fig. 1*B*). This allows the soft millirobots to retain the soft-bodied locomotion strategies previously developed for precise positioning and orientation. Design strategies to optimize the performance of the anchoring and triggering mechanism based on simulations are also presented. We demonstrate the anchoring mechanism on a variety of ex vivo tissues. Finally, we demonstrate control of the duration of anchoring and realize subsurface drug delivery using needles made from a biodegradable material and hollow needles, respectively, showcasing the versatility of the proposed mechanism.

## Results

### Working Principle.

The proposed mechanism is a spring-loaded mechanism encapsulated in a casing and is composed of three components: a barbed needle mounted on a copper plate for insertion and anchoring, a preloaded spring to provide the required force output, a casing to enclose the structure, and a thermal-degradable adhesive which holds the copper plate in the casing and acts as a trigger (Fig. 1*C*). As we intended to integrate the mechanism with magnetic soft millirobots, copper was selected as the heating element because it was nonmagnetic and would not interfere with magnetic actuation of the robots. Stainless steel needles with a shaft diameter of 100 µm were used, and barbs were added to enhance the anchoring force. An additional advantage of using springs was the rapid release of energy along the axis of compression, which could accelerate the needle to very high velocities during release. Based on high-speed video image snapshots, this velocity is estimated to be at least 50 mm/s. As it has been previously reported that the penetration force in ex vivo porcine heart tissues is inversely proportional to the impact velocity (39), a needle with a speed of 50 mm/s requires ∼50% less puncture force than one traveling at 5 mm/s.

The inner diameter of the casing encapsulating the spring was designed to be 0.1 mm larger than the diameter of the copper plate. For the copper plate and casing used in this work, the diameters were 2.0 and 2.1 mm, respectively. Due to this difference in size, the copper plate would initially be unable to stop the motion of the spring. However, by applying a thin layer of cyanoacrylate adhesive to the sides of the copper plate, the diameter of the copper plate was increased such that it was slightly larger than the diameter of the casing. After the glue had completely cured, the plate was then press fitted on the casing, encapsulating the spring within the casing. To trigger the mechanism (Fig. 1*D*), an external 338-kHz RF field was applied. From the Faraday’s and Lenz’s law, the RF field induced an eddy current inside the copper plate such that it produced a magnetic field that opposed the direction of the applied field (40). The eddy current heated the copper plate via Joule heating and heated up the surrounding layer of cured adhesive via thermal conduction. When the temperature reached 60 °C, the strength of the cured adhesive was reduced by 40% compared to the strength at room temperature; the adhesive layer was no longer able to hold back the compressed spring (41). At this point, the spring was free to extend and pushed the copper plate together with the needle toward the surface (Fig. 1*E*). We verified the temperature triggered mechanism by taking infrared images of the triggering process (Fig. 1*F*).

### Anchoring Performance of the Mechanism.

**Fig. 2.**
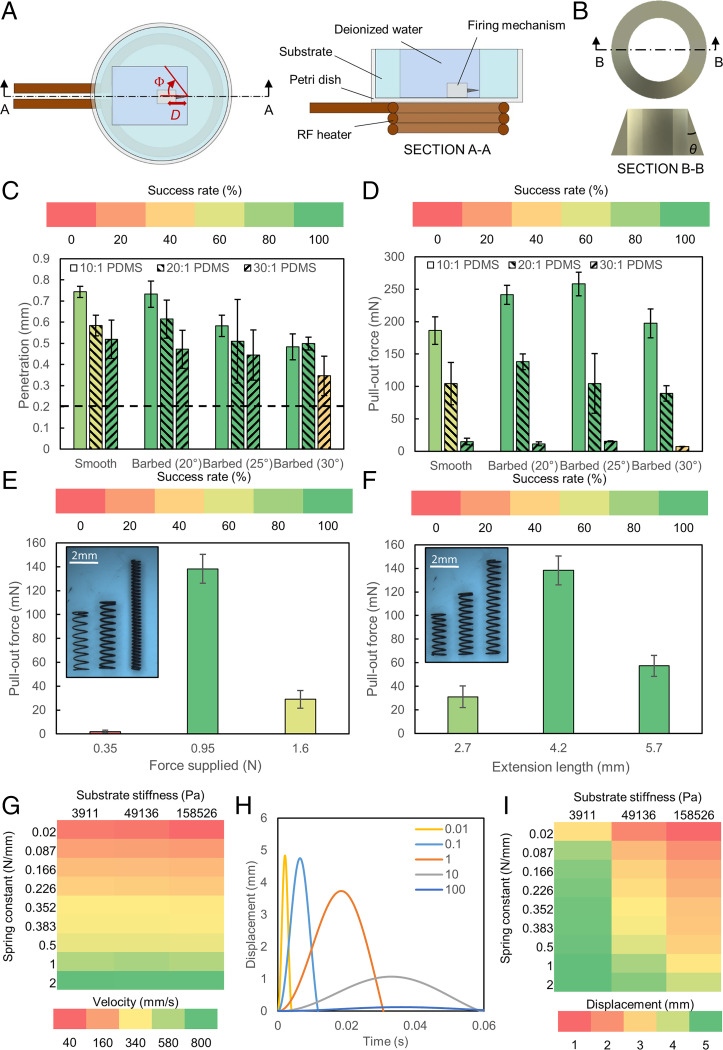
Mechanism characterization. (*A*) Schematic of experimental setup. (*B*) Schematic of barb design. (*C*) Penetration depth with respect to the different substrates used in this work (*n* = 5). The dotted line denotes the position of the barb. Error bars represent the SD. (*D*) Pull-out force of different barb designs with respect to the different substrates (*n* = 5). Error bars represent the SD. (*E*) Pull-out force with respect to the force supplied by the spring (*n* = 5). Error bars represent the SD. (*F*) Pull-out force with respect to the extension length of the spring (*n* = 5). Error bars represent the SD. (*G*) Velocity at 1 mm with respect to substrate stiffness and spring constant at a mass ratio of 1. (*H*) Displacement–time graph of the mass $m_{1}$ at different $\frac{m_{1}}{m_{2}}$ mass ratios traveling into the substrate during phase I. The colors represent the different mass ratios. (*I*) Maximum displacement with respect to substrate stiffness and spring constant at a mass ratio of 1.

The performance of the mechanism was assessed based on two parameters: success rate and pull-out force. A successful event was defined as the case where the needle remained attached to the surface after remote triggering. The pull-out force was the peak force recorded as the needle was removed from the surface. The prototypes were submerged in deionized (DI) water and were not fixed to the substrate in all the experiments (Fig. 2*A*). To find the optimum design of the anchoring mechanism, we categorized the performance based on two design groups: barb design and spring selection. In the former, the effects of adding a barb on the performance on different surfaces were evaluated. In the latter, since the spring was one of the critical components of the mechanism, the effect of using different springs was examined. Polydimethylsiloxane (PDMS; Sylgard 184, Dow Corning) at base to curing agent mass ratios of 10:1, 20:1, and 30:1 were selected as the soft surfaces for the characterization tests because the Young’s modulus of these surfaces (*SI Appendix*, Fig. S1) fell within the range of the most biological tissues (42), and the Young’s modulus was the dominant mechanical property during the prepuncture stage (43).

First, we investigated how the geometry of the needle barb could affect the anchoring performance. Due to the asymmetry of the barbs in the axial direction (Fig. 2*B*), the needle was also able to utilize mechanical locking with the surface, in addition to the friction between the embedded needle and the surface, for anchoring. A barbed needle resulted in a lower penetration depth since more energy was required to drive the needle into the surface to the same depth as a barbless needle (Fig. 2*C*). This decreased the length of the needle embedded in the substrate after anchoring and, hence, the frictional force. However, this reduction in anchoring force was less dominant than the increase brought by the mechanical locking between the barbs and the surface. This is reflected in the form of an increased pull-out force (up to 38%) and success rate (Fig. 2*D*). Furthermore, as the stiffness of the surface decreased, it was observed that the cone angle at which the maximum pull-out force was recorded also decreased. At a given cone angle for the barbs, a surface with a lower stiffness would be able to deflect more, requiring the needle to travel deeper into the surface before the stress induced at the needle tip exceeded the critical stress required for puncture. Note that the critical stresses required for puncture are similar throughout all the substrate samples because of their similar fracture toughness (*SI Appendix*, Fig. S1). The increased deformation of a softer surface explains why a shorter length of the needle was left embedded in the surface (Fig. 2*C*). This shorter penetration length resulted in a lower pull-out force as the stiffness of the surface decreased. Hence, a smaller cone angle should be used on a surface that is less stiff in order to achieve the largest possible pull-out force.

Next, to investigate the effect of changing the spring parameters on the performance, we conducted two sets of experiments on a 20:1 PDMS surface using a barb with a cone angle of 20°. Springs, which were suitably small (<5 mm in both diameter and compressed length) but could still provide almost 1 N of force, were used in these tests, to facilitate integration with miniature magnetic soft robots. We first selected a set of springs by varying the maximum amount of the force while keeping the extension length constant (Fig. 2*E*). Next, we changed the maximum extension length of the springs (Fig. 2*F*) while keeping the maximum force constant. Both results indicated that there was an optimal value in which the largest pull-out force could be achieved. By combining and reexpressing the figures with respect to the spring constants, we could see that the spring with a medium spring constant 0.226 N/mm out of the five tested springs (with spring constants ranging from 0.087 to 0.383 N/mm) gave the highest anchoring success rate and the largest pull-out force (*SI Appendix*, Fig. S2). To better understand this, an analysis of the anchoring mechanism was conducted. Details of the analysis are provided in *SI Appendix*, *Analysis of the Mechanism* and Fig. S3.

From the analysis, we observed that the velocity of the needle after traveling 1 mm distance into the tissue was almost independent of the Young’s modulus of the surface and was largely dependent on the spring constant (Fig. 2*G*). Given that the puncturing force would be the same, since the fracture toughness of the surfaces was similar and the needle length was sufficiently short (1 mm) such that the difference in kinetic friction did not have an appreciable effect on the speed, it can be inferred that the copper plate mounted on a spring with a larger spring constant would have a larger impact and higher exit velocity. As a result, the needle would move back out of the substrate after penetration. This explains why an optimal value for the spring constant for a particular substrate exists (Fig. 2 *E* and *F*). The spring must be able to provide enough force to drive the entire length of the needle into the substrate and yet, dissipate enough energy during the process so that the remaining energy would be smaller than the energy required to pull out the needle.

Although the spring constant is highly dependent on the fracture toughness of the surface, a spring with a larger spring constant should be used as the fracture toughness of the surface increases or if a longer needle is used since more energy will be used to overcome friction during the insertion phase. Should the exact fracture toughness be unknown, as in the case of most biological samples, we recommend that a more conservative value (i.e., spring with a lower spring constant) first be used. From the results presented in *SI Appendix*, Fig. S2, we observe that using springs with overly high constants (0.352 and 0.383 N/mm) results in lower success rates and pull-out forces in comparison to springs with more conservative spring constants (0.166 and 0.226 N/mm).

Two other conclusions can be made about the system from the analysis. First, although distributing more mass to the casing is favorable in achieving higher penetration, this ratio cannot be increased infinitely and must be balanced against the RF heating efficiencies. We observed that the copper plate and needle with a combined mass of $m_{1}$ would be able to move deeper into the surface as more mass was distributed to the casing, $m_{2}$, which increased the likelihood of penetration and anchoring. Moreover, as the mass ratio $\frac{m_{1}}{m_{2}}$ decreased, the velocity of $m_{1}$ also increased (Fig. 2*H* and *SI Appendix*, Fig. S4). However, as the mass ratio drops, the thickness of the copper plate will decrease given a fixed diameter. Correspondingly, this will reduce the heating efficiency and hence the triggering performance of the mechanism. As an example, when the mass ratio is 0.1, the thickness of the copper plate will become 70 µm given the current geometry. This is lower than the skin depth of the copper, which is calculated to be 117 µm at 338 kHz (44). At thicknesses below the skin depth, RF heating efficiency will be compromised, and the required temperatures might not be achievable, or it might require extended heating durations. Please refer to *Performance of the Anchoring Mechanism in Untethered Robotic Systems* for an analysis on RF heating of the system. Therefore, a mass ratio of 1 was used in this work as it was the lowest mass ratio that could be used without compromising the heating efficiency of the mechanism.

Second, a longer needle is always superior to a shorter needle. From the simulations, we observed that a surface with a lower Young’s modulus was able to deform more (Fig. 2*I*). Therefore, at a given load, the contact area between the needle tip and the surface will be larger for a surface with a lower Young’s modulus (*SI Appendix*, Fig. S5). This in turn lowers the stress concentration at the needle tip, and as such, the needle will have to translate deeper into the tissue in order to achieve puncture. Compared to shorter needles, longer needles can achieve a higher stress concentration as they are able to translate deeper into the substrates and allow for puncture even on softer substrates. Moreover, a longer needle increases the duration of the needle insertion phase (*SI Appendix*, Fig. S3*B*). A longer insertion phase allows for more kinetic energy to be dissipated by friction as the needle travels through the surface after puncture, which in turn reduces the impact and exit velocity after the copper plate impacts the surface. This allows a longer length of the needle to remain embedded in the surface, translating to a larger pull-out force. As such, if overpuncturing is not a concern in the design, a spring with the highest spring constant within the design limits should be used in tandem with a long needle, with the upper limit defined by the critical buckling load of the needle.

### Performance of the Anchoring Mechanism in Untethered Robotic Systems.

**Fig. 3.**
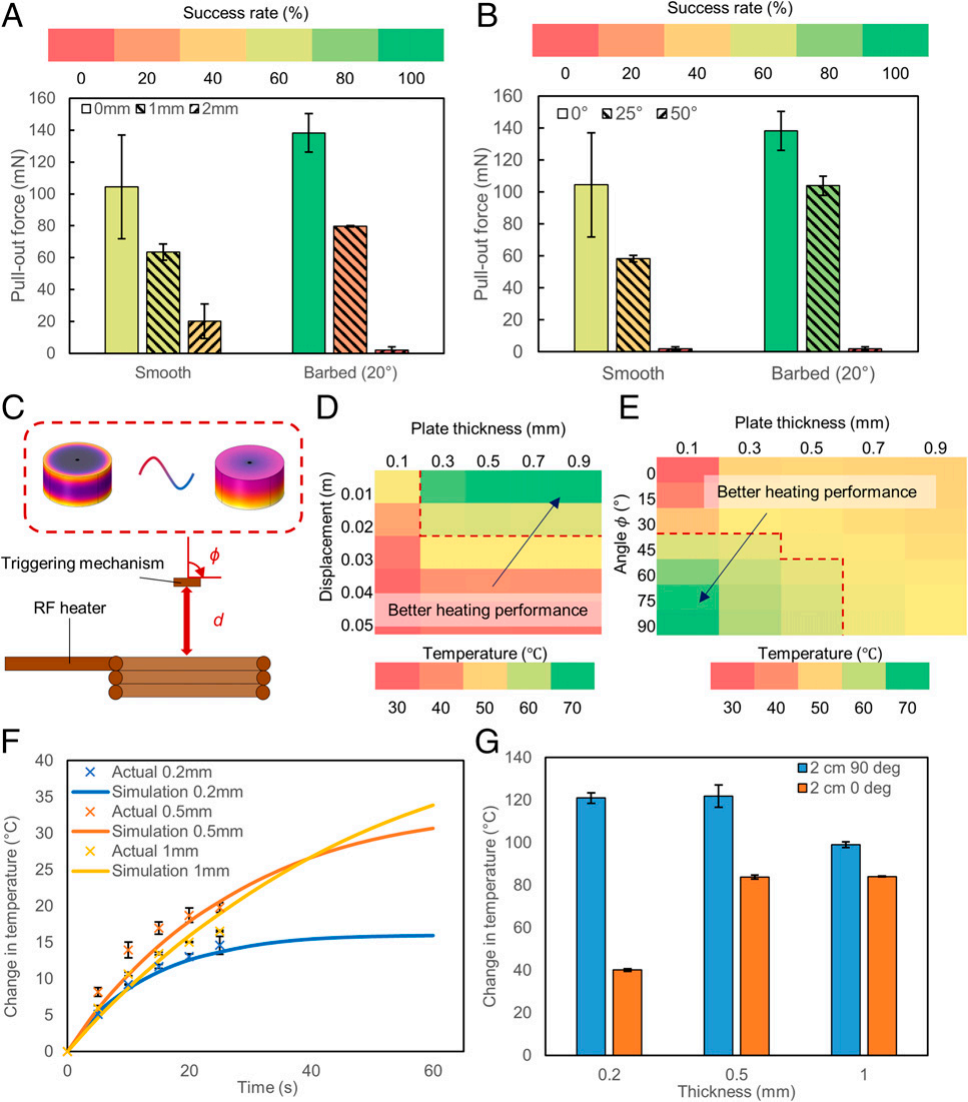
Mechanism characterization and modeling. (*A*) Pull-out force with respect to the distance of needle from the substrate (*n* = 5). Error bars represent the SD. (*B*) Pull-out force with respect to the angle of approach to the substrate (*n* = 5). Error bars represent the SD. (*C*) Schematic of experimental setup for heating experiments. (*D*) Predicted temperature of the copper plate after 25 s with respect to plates of varying thicknesses and distances at a constant angle *ϕ* = 0°. The red dotted line indicates that the temperature has exceeded 60 °C. (*E*) Predicted temperature of the copper plate after 25 s with respect to plates of varying thicknesses and angles at a constant distance of d = 0.02 m. The red dotted line indicates that the temperature has exceeded 60 °C. (*F*) Temperature rise of copper plate of varying thicknesses (t = 0.2, 0.5, and 1.0 mm) predicted from finite element simulation plotted against experimental values at d = 0.03 m, *ϕ* = 0° (*n* = 3). Error bars represent the SD. (*G*) Difference in temperature due to a change in angle at d = 0.02 m (*n* = 3). Error bars represent the SD.

As we could accurately control the position of the mechanism by integrating it with wireless magnetic soft robots, we investigated the effects of distance and orientation on the anchoring and heating performance of the mechanism. First, the effects of parameters which could be controlled externally by a robot such as changing the distance away from the surface, *D*, and orientation, Φ, were investigated (Fig. 2*A*). It was observed that as the distance between the needle tip and the surface increased, the recorded pull-out force decreased (Fig. 3*A*). Eighty percent of the tested barbed needles, which were able to achieve the highest pull-out force previously, did not successfully penetrate the surface when the distance between the surface and the needle tip (i.e., *D*) was increased to 1 mm. The total extension of the spring used in this test was 4.2 mm. Similarly, at increasing Φ (Fig. 3*B*), we recorded a drop in the performance of the mechanism. The decrease in performance can be explained by the decrease in the applied force. In the former, as the distance between the needle tip and the surface increased, the force which was applied by the spring to deform the surface decreased as the spring had extended more. In the latter, as some of the resultant force was directed to translate the needle parallel to the surface, less force was available to drive the needle into the surface. As such, in order to achieve higher pull-out force and thus better anchoring, it is better to place the mechanism closer and/or perpendicular to the surface.

Next, we investigated the effects of distance and orientation on the maximum heating achieved by the copper plate (Fig. 3*C*). As the distance between the copper plate and the RF heater, *d*, increased, the amount of generated heat decreased, indicated by the lower recorded temperature after 25 s of RF exposure. This was due to a rapid decay of the alternating magnetic field which induced less current density and, hence, decreased the rate of heating of the copper plate. As a result of this reduced rate of heating, the temperature rise became independent of the thickness of the copper plate. For instance, based on the simulations, although there was a substantial difference in temperature of over 70 °C between the 0.9-mm-thick plate and the 0.3-mm-thick plate at a distance of 0.01 m, the temperature difference was less than 1 °C at a distance of 0.03 m (Fig. 3*D*). We validated the simulations by comparing the results obtained from the experiments with those obtained from the simulations (Fig. 3*F*). As such, to reach the required temperatures at larger distances, the RF field is applied for longer durations or at higher frequencies and amplitudes. From the simulations, we also find that increasing the input current amplitude is a more effective strategy as compared to increasing the frequency of the RF field (*SI Appendix*, Fig. S6).

Without increasing the input current, frequency, or duration of RF exposure, one could also increase the heating rate by changing the orientation of the heater with respect to the triggering mechanism. In this regard, the major axis of the heating plate was aligned such that it was parallel to that of the RF coils (ϕ = 90°). In doing so, the effective distance could be increased by 75% to 3.5 cm (Fig. 3 *E* and *G*). This would be sufficient for use in organs directly under the abdominal skin, which has an average thickness of 2.6 to 3.1 cm (45), such as the bladder. This can be further increased as the diameter (i.e., volume) of the heating plate is increased (*SI Appendix*, Fig. S7), although it has to be balanced against limits imposed by the intended operating environment. For instance, a 0.1-mm-thick heating plate with a diameter of 4.5 mm, which could be deployed in the bladder (46), was able to reach a temperature of 60 °C at a distance of 5 cm in our experiments. Due to the constraints imposed by the experimental setup, all the experiments in this work were conducted such that the copper plate was perpendicular to the RF coils (ϕ = 0°), which limited the maximum heating which could be achieved. As such, it was necessary to place the device at a very close distance from the RF coils. However, if the magnetic coils used for actuation are also able to give a high-frequency oscillating field for remote heating, then a working distance of 3.5 cm can be achieved with the current setup.

To further extend the range of the device, insulating layers can be added to the heating plate to minimize the heat dissipated to the surrounding environment. For instance, when a thin insulating polyimide layer of 50 µm was added to the outer surface of the copper plate (side exposed to DI water), the temperature rise on the surface was reduced by over 20 °C (*SI Appendix*, Fig. S8), indicating that more of the generated heat was directed to the side with the adhesive. This not only allows the device to operate at a longer distance but also protects the tissues from being heated by the copper plate during triggering.

### Proof-of-Concept Demonstrations.

**Fig. 4.**
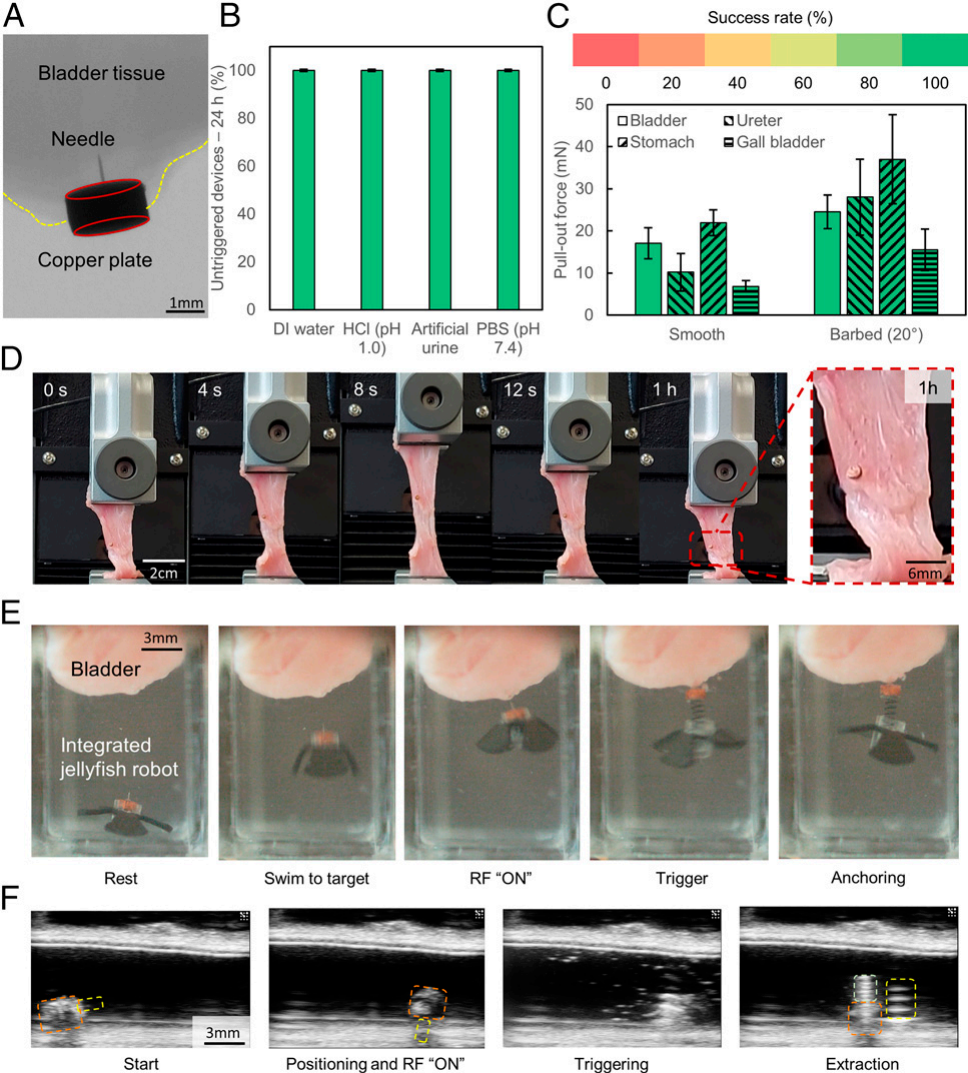
Demonstration of the proposed anchoring mechanism. (*A*) Micro-CT image showing anchoring of the device on an ex vivo bladder tissue. The yellow and red lines represent the boundary of the bladder tissue and the orientation of the copper plate, respectively. (*B*) Robustness of the mechanism in various simulated body fluids (*n* = 5). Error bars represent the SD. (*C*) Pull-out force of the needle on biological tissues (*n* = 5). Error bars represent the SD. (*D*) Experiment to simulate dilation and relaxation of the bladder. (*E*) Mechanism integrated with a jellyfish robot for anchoring in fluid-filled three-dimensional spaces. (*F*) Ultrasound images of the mechanism integrated with a sheet-shaped soft robot for anchoring in confined spaces. Orange, yellow, and light green outlines represent the casing, needle mounted on copper plate, and spring, respectively.

As proof-of-concept demonstrations, we tested the anchoring capabilities of our mechanism-integrated soft millirobots in various simulated ex vivo environments. To verify if the needle had indeed achieved successful penetration of the tissue, we deployed the mechanism on an ex vivo porcine bladder and took microcomputed tomography (micro-CT) images (Fig. 4*A*). Given that there are constant dynamic motions and a variety of fluids inside the human body, we then tested the robustness of the triggering mechanism. In this regard, we submerged the mechanism in simulated body fluids and environments and observed if they could be accidentally triggered. Fig. 4*B* shows that the device was stable over a period of 24 h over a variety of simulated body fluids, highlighting the robustness and insensitivity of the triggering mechanism to biological fluids. Fig. 4*C* shows the pull-out force required in organs found in the urinary and gastrointestinal tract. Even though there was a drop in the pull-out forces as opposed to the surfaces used in the characterization tests, the effect of the barb in enhancing the anchoring force was still evident. The decrease in anchoring force can be primarily attributed to the increase in the fracture toughness of biological tissues (e.g., muscles, cartilages, and connective tissues) (47), which is measured to be on the order of 1 kJ/m^2^ (48–51) as compared to 0.3 kJ/m^2^ for PDMS (*SI Appendix*, Fig. S1). The recorded force was also comparable to those recorded by the barbed needles in the literature (*SI Appendix*, Table S3).

We then applied cyclic extensional strains to the ex vivo bladder tissue after successful anchoring of the mechanism onto it. Even after the bladder tissue was subjected to a strain of 100% for 100 cycles over an hour, we observed that the needle remained firmly attached to the surface of the bladder, indicating the robustness of the anchoring performance (Fig. 4*D* and Movie S1). Next, we integrated the mechanism with a jellyfish-inspired soft millirobot to allow for accurate navigation inside open fluid-filled environments such as the bladder. The jellyfish robot was able to carry the anchoring mechanism, trigger it while it was still swimming, and perch itself onto an overhanging bladder tissue (Fig. 4*E* and Movie S2). On the other hand, for procedures in confined spaces inside the body, such as the ureter and urethra, we incorporated the mechanism with a conventional sheet-shaped soft millirobot. The mechanism was also compatible with existing medical imaging techniques, such as ultrasound imaging (Fig. 4*F* and Movie S3).

These demonstrations highlight the potential of integrating the proposed anchoring mechanism on various wireless soft millirobots. In all of these demonstrations, the RF field was turned on for less than 1 min to trigger the anchoring mechanism. To ensure that there was minimal heating of the tissue, we exposed bladder tissues to the same field for 1 min and observed that there was a negligible increase (<0.2 °C) in temperature (*SI Appendix*, Fig. S9). We also anticipate minimal nerve and muscle stimulation, which occurs at frequencies below 10 kHz (52). The applied 338-kHz field is also close to those provided by electrosurgical units in use by hospitals today (500 kHz to 3 MHz) (53).

### Multifunctional Needle toward Medical Applications.

**Fig. 5.**
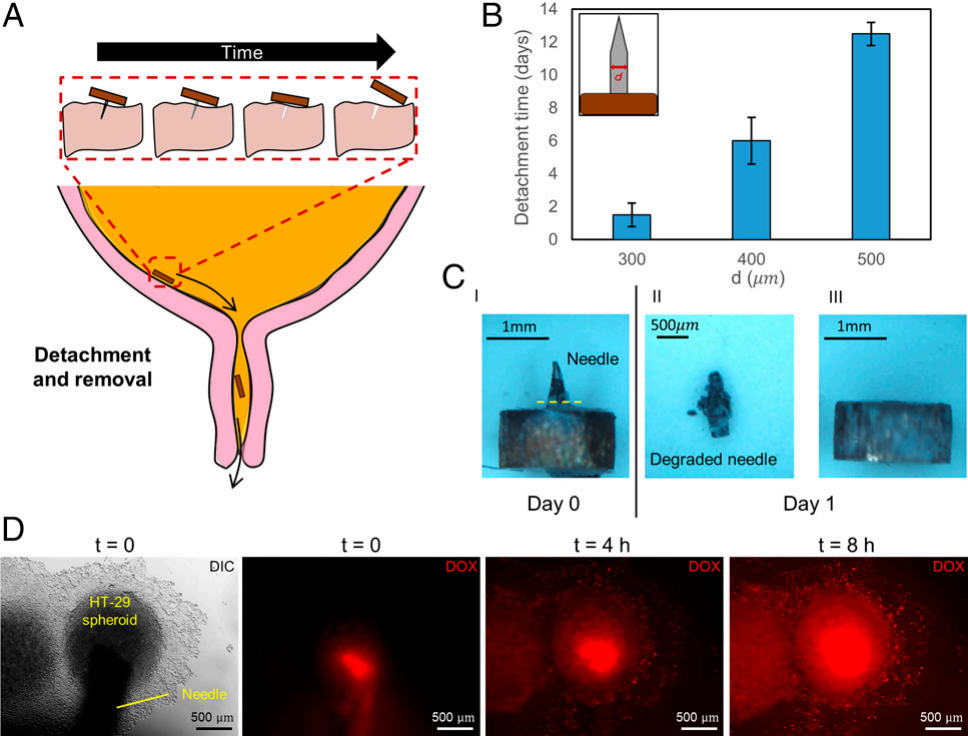
Multifunctional needle toward medical applications. (*A*) Conceptual illustration of controllable detachment and passive removal of the device after anchoring. (*B*) Time to detachment of copper plate from the substrate using biodegradable needles of different diameters (*n* = 2). Error bars represent the standard deviation. (*C*) The 300-µm-diameter biodegradable needle before and after degradation. (*C*, *I*) Image taken on day 0 before it was anchored on a 10:1 PDMS substrate. The yellow line indicates the degradation site. (*C*, *II*) Close-up photo of the needle. The needle was removed from the PDMS substrate for imaging. (*C*, *III*) Detached copper plate. Images in *C*, *II* and *C*, *III* were taken on day 1, after the structure had been submerged in DI water in an incubator at 37°C. (*D*) DOX delivery to HT-29 spheroids. The needle was punched into the HT-29 spheroids, and time lapse images were recorded. The fluorescence signal of DOX distributed in the spheroid in time, demonstrating the drug release capability of the needles.

Last, we demonstrate how additional functionalities, such as controlled detachment and subsurface drug delivery, can be incorporated simply by changing the needle type. In the former, we fabricated needles of different diameters with a bioresorbable magnesium-based alloy (Resoloy, MeKo Manufacturing e.K.) and anchored them on a 10:1 PDMS substrate. The needles and substrates were then placed in an incubator set at 37 °C to simulate the temperature of the human body. Over time, the magnesium alloy hydrolyzed and degraded, allowing the copper plate, which was initially attached to the needle, to detach from the anchored PDMS surface. By controlling the diameter of the needles, the detachment time could be altered from 1 d to almost 2 wk (Fig. 5*B*). Degradation primarily occurred along the needle that was not embedded in the surface and exposed to water (Fig. 5*C* and *SI Appendix*, Fig. S10). Such a function might allow for passive retrieval of the anchored components—for long-term in situ sensing or sample collection—at a specific time. For example, since the diameter of the copper plate used in this work is 2 mm, the copper plate and the other components can be passively expelled from the body if the device is deployed in the bladder (Fig. 5*A*). We do not anticipate any problems during this process as kidney stones with diameters less than 5 mm can already be spontaneously expelled by the body (46). Results from the initial biocompatibility tests for long-term anchoring are presented in *SI Appendix*, *Initial Biocompatibility Studies* and Fig. S11.

In the latter, we used hypodermic needles loaded with model drugs to demonstrate subsurface drug delivery. Drug delivery to most tumor sites through the vascular system is highly ineffective due to factors such as a high interstitial tumor pressure or a leaky blood vessel (54). As such, delivering drugs directly to the tumor site could potentially enhance therapeutic effects while lowering the required dose. Here we demonstrate initial steps toward a functional device for in situ drug delivery. By switching the solid needle with a hollow needle and preloading a model cancer drug, doxorubicin (DOX), into the needle, we were able to deliver DOX directly inside a tumor spheroid through diffusion (Fig. 5*D* and Movie S4).

## Discussion

Abramson et al. (55) demonstrated a spring-loaded mechanism that could provide the necessary force to deliver a barbed needle into the soft tissue surface. However, there was no active control over the release time and the position and orientation of the device once it was swallowed. Moreover, the needle release mechanism was passively triggered by changes in the environment, making it highly dependent on and specific to the local environmental conditions. In this work, we demonstrate a mechanism which can be wirelessly triggered to generate the high normal forces required to drive a needle into a specific location on a soft surface for on-demand anchoring. By placing a barb on the needle, the pull-out force was increased by up to 38%, thereby enhancing the long-term anchoring capability of the mechanism. Guidelines to optimize the triggering and anchoring performance of the proposed mechanism were established. Most importantly, the mechanism was small and light enough to be mounted on existing soft magnetic millirobots. The locomotion and anchoring capabilities of these integrated robots on ex vivo tissues were then demonstrated. Initial steps toward functional medical devices demonstrating controlled detachment and drug delivery were achieved by using bioresorbable needles with different diameters and hypodermic needles, respectively. Overall, these functionalities highlight the possibility of using such untethered robots in medical applications.

For successful translation, future studies should concentrate on three aspects. First, the safety of an exposure to RF fields should be assessed with in vivo experiments. Although International Commission on Non-Ionizing Radiation Protection (ICNIRP) provides general guidelines, these restrictions can be exceeded in application-specific instances, such as RF hyperthermia for cancer therapy (56), and are commensurate with the risk and benefit to the patient. However, in cases where such an exposure is not advisable, focused ultrasound can be used to heat up the triggering mechanism instead (57). Second, we note that all the materials used in the fabrication of the mechanism have biocompatible equivalents, which are being used in devices designed for long-term use inside the human body (*SI Appendix*, Table S4). Nonetheless, histological studies to verify the safety of the heated plate impacting the tissue surface and the immune response of the tissue to the long-term anchoring of a foreign device should be conducted before in vivo experiments are performed to minimize pain to the animals. Last, future studies should investigate the possibility of incorporating additional functions. For instance, in the drug delivery demonstration, hollow needles can be fabricated using other biodegradable materials with specific degradation mechanisms. An example of such material is silk fibrin, which can be degraded enzymatically by protease (*SI Appendix*, Fig. S12). Having this design flexibility will provide more options for specific target medical applications. Addressing these issues would further enhance the capabilities of this mechanism and can potentially enable a range of minimally invasive long-term medical procedures, which are currently unavailable.

## Materials and Methods

### Wireless Magnetic Soft Millirobot Fabrication.

The soft millirobots were fabricated with a method described previously (10). Briefly, Scotch tape (840, 3M) was first placed along the sides of an acrylic sheet. The number of layers placed determined the thickness of the robot. Neodymium-iron-boron (NdFeB) magnetic microparticles (MQP-15–7, Magnequench) were mixed with platinum-catalyzed silicone (Ecoflex 00-10, Smooth-On Inc.) in a 1:1 mass ratio, degassed for 5 min, poured on the acrylic sheet, and evened out with a razor blade. The sample was then left to cure on a hot plate at 65 °C for 1 h. After the sample had cured, it was laser cut (ProtoLaser U3, LPKF Laser & Electronics AG) to the desired dimensions. The structures were then detached from the substrate with tweezers and wrapped around a nonmagnetic sphere (diameter of 5 mm) or rod (diameter of 3.2 mm) for the jellyfish and sheet robot, respectively. The robots were then magnetized with a 1.8 T homogeneous magnetic field in a vibrating sample magnetometer (EZ7, Microsense) for 5 s. Water-soluble glue (822095, Pritt) was applied during this process to ensure that the robots tightly conformed to the magnetization structures. The nonmagnetic sphere and rod were printed on a stereolithography printer (Stereolithography (SLA), Form 3, Formlabs) with the Clear Resin. Next, the robots were detached from the structures and rinsed with DI water until no more glue was observed on the surface. Last, the preassembled firing mechanisms were attached to the robots with cyanoacrylate glue (401, Loctite). Whenever necessary, a stereomicroscope (ZEISS Stemi 508, Carl Zeiss Microscopy GmbH) was used to guide the process.

### Firing Mechanism Fabrication.

Springs (CBM010B04E, CBM014A09E, CBM014B07E, CBM014B02E, and CBM014B05E, Lee Spring) and needles (S-J1015, Seirin) were used as purchased. The 2-mm-diameter copper rings for the heating element were laser cut from a 1-mm-thick copper plate. A 0.11-mm opening was also laser cut at the center of the ring to mount the needles. The needles were then marked at a distance of 1 mm away from the needle tip with a marker. Next, the needles were threaded through the copper plate. Once the 1-mm distance mark appeared on the other side of the copper plate, cyanoacrylate glue (401, Loctite) was applied to fix it in position on both sides of the copper plate. After the glue had set, the needle was snipped off with a pair of wire cutters. As it was impossible to flush the cutting edge of the wire cutters to the surface of the copper plate, a small length of the needle would still remain on the copper plate after snipping. The extra needle was folded inward, and cyanoacrylate glue (401, Loctite) was used to fix it to the surface of the copper plate.

If required, the barbs were added at this stage. The barbs were fabricated using two-photon polymerization with a negative-tone photoresist (IP-S, Nanoscribe GmbH). They were positioned such that they were 0.2 mm away from the needle tip. All procedures involving any measurements are performed by hand under a stereomicroscope using a stage micrometer as a guide (R1L3S1P, Thorlabs). The casing and a holding jig were printed using the Clear Resin with the above-mentioned resin. Next, cyanoacrylate glue (431, Loctite) was applied to the side of the copper plate. After curing on a hot plate at 40°C for 2 min, the copper plate with the needle was assembled with the casing and spring. One end of the spring was secured to the casing with cyanoacrylate glue (401, Loctite). The casing was printed on a stereolithography printer (SLA, Form 3, Formlabs) with the Clear Resin. On the other end of the spring, the copper plate was first placed on top with the needle pointing outward and then pushed into the structure.

### Characterization of the Firing Mechanism.

The PDMS substrates were prepared in the required ratios (10:1, 20:1, and 30:1). Five grams of the samples were poured into a 55-mm diameter Petri dish. After curing in an oven at 90°C for 2 h, an area measuring ∼3 cm × 3 cm was removed from the center and filled with water. The firing mechanism was then submerged and placed in the appropriate location. The entire Petri dish was then placed at a distance of 3 mm above the RF heater probe, with the firing mechanism visually positioned to be at the center of the probe. The RF heater was set at 751.8 A at a frequency of 338 kHz. All of the characterization tests were recorded with a high-speed camera (Miro M310, Phantom) at 2,600 frames per second (fps).

The penetration depth was determined by postanalysis of the high-speed videos on ImageJ. The length of the needle not inside the surface was subtracted from the initial length of the needle (1 mm in this work) to reduce problems associated with refraction. To measure the pull-out force, a pair of tweezers was clamped to the end of an Instron machine (5942, Instron) with the 5 N load cell while the surface was stuck to the other. Tweezers were used to reduce the contact area to make gripping of the copper plate possible. The pull-out force was defined as the highest force recorded.

### Characterization of the Copper Heating Plate.

Copper plates with thicknesses of 0.2, 0.5, 0.8, and 1.0 mm were cut into the required dimensions. Temperature measurements were taken with an infrared camera (ETS320, Teledyne FLIR). The copper heating plate was first positioned visually at the center of the RF coil at the appropriate height. As the camera has a fixed focus distance of 7 cm, the infrared camera was placed 7 cm above the copper plate during the experiments. Before the tests, the camera was calibrated with melting ice and boiling water. As copper has a low emissivity and could affect the accuracy of the readings, a 50-μm Kapton layer (KAP22-075, Thorlabs) was attached to the surface. Data were collected either once every 5 s for 25 s or once at 25 s; 25 s was chosen because it would generate five data points, which could then better describe the nonlinear trend.

### Finite Element Analysis.

Simulation results presented in Fig. 3 were generated with the commercially available finite element software, COMSOL Multiphysics. The RF heater was modeled as a homogenous multiturn coil as per the dimensions and values provided by the manufacturer. External natural convection and radiative heat transfer were implemented along the boundaries as heat losses. The mechanical and electrical properties of the materials provided by COMSOL were used without further modification.

### Surface Young’s Modulus Measurement.

Young’s modulus measurements were adapted from the D412 ASTM (American Society for Testing and Materials) standard. A sample with dimensions corresponding to Die C, with a 3-mm thickness, was prepared. The samples were tested at a rate of 500 mm/min up to a strain of 0.50. The Young’s modulus subsequently was computed by fitting a straight line to the stress–strain curve up to a strain of 0.25. Each sample was tested three times.

### Fracture Toughness Measurement.

Fracture toughness measurements were adapted from a previous publication (58). A sample with dimensions 75 mm × 50 mm × 3 mm was prepared and cut into two equal 75 mm × 25 mm × 3 mm samples. A 20-mm notch was then made on one of the samples with a surgical scalpel. The unnotched sample was then clamped and stretched to get the force–length curve. It was unnecessary to pull this sample to failure. The notched sample was then clamped and pulled until the notch turned into a running crack. The length *L* at which this occurred was visually determined by recording the process with a high-speed camera at 30 fps. The fracture energy was then calculated by finding the area under the force–length curve of the unnotched sample at *L* and dividing it by the width and thickness (75 mm and 3 mm, respectively). All samples were clamped such that the distance between the clamps was 5 mm. All the samples were stretched at a strain rate of 0.5/min.

### Friction Measurements.

Friction measurements were adapted from the D1894 ASTM standard. A sled wrapped with foam was first weighed to determine the normal force. Next, a 2-cm^2^ sample (PDMS, Dow Corning) of 0.5 mm thickness was attached with double-sided tape (56665, Tesa) to the sled. The sample was visually positioned to be in the middle of the sled. A nylon tow line was then used to connect the sled and the 5 N load cell. The sample and test surface were cleaned with propan-2-ol, and the load cell was translated upward to ensure that the tow line was taut. The force and displacement readings were then zeroed before the load cell was translated at 10 mm/min for 15 mm. The initial peak force was recorded as the static frictional force. As the sled began to translate on the test surface, there was an immediate drop in the force recorded. The kinetic frictional force was obtained by calculating the average force value from which the force began to rise again after the first initial drop to the end of the run. The coefficient of static and kinetic friction was then calculated by dividing the respective forces by the weight of the sled. Three samples were tested for each substrate. The samples were tested on a universal load machine (5942, Instron) equipped with the friction fixture.

### Ex Vivo Proof-of-Concept Experiments.

Pig organs from a slaughterhouse were obtained and stored at 5°C. Organs used in this work were the urinary bladder, ureter, stomach, and gall bladder. The pull-out forces presented in this work were obtained in the same fashion as the characterization tests. In this regard, an organ replaced PDMS as the surface. The organs were tested within 24 h upon receiving. All experiments were performed in DI water. For the stretching experiments, a 100% strain at a rate of 100 cycles per hour was applied to a 3 cm × 1.5 cm strip of bladder after a needle was anchored on it with the mechanism. For the jellyfish demo, the surface was substituted with a 2 cm × 2 cm bladder piece. A single-axis magnetic coil was used to apply the magnetic fields required for actuation. The coils were controlled with a custom-written LabVIEW code. The waveform used to actuate the jellyfish robot is presented in *SI Appendix*, Fig. S13. In both demos, the mechanisms were anchored on the interior surface of the bladder. In the ultrasound-guided demonstration, the robot was positioned using a handheld magnet. Similar to all the characterization experiments, an RF field at 751.8 A with a frequency of 338 kHz was used to trigger the mechanism. The RF field was acting on an axis perpendicular to that used for magnetic actuation.

### Durability Tests.

Assembled devices were placed in each glass vials containing 6 mL of liquid and a magnetic stirrer. The liquids used were PBS, pH 7.4 (1×) (10010-023, Gibco), DI water, artificial urine (30401, Sun-Vi), and hydrochloric acid, pH 1.0 (35642, Alfa Aesar). Following that, they were placed on a hot plate (IKA RCT basic, Lab Logistics Group (LLG) Labware) at 37 °C. The stirrer was set at 100 revolutions per minute. The number of untriggered devices after 24 h of continuous stirring at 37 °C were counted.

### Bioresorbable Needle Fabrication.

A 0.9-mm-diameter magnesium alloy was purchased from MEKO, cut into 3-cm sections and machined into needles on a lathe to the required diameters. All needles had a cone angle of 10°. The needles were then assembled and tested as per the methods stated above (*Firing Mechanism Fabrication* and *Characterization of the Firing Mechanism*). For the cell culture experiments, the material was made into a powder by filing the raw material.

### Hollow Needle Fabrication.

Cotton wool was inserted by hand into hypodermic needles (23G, Terumo Agani). The sharp edge was filed down to ensure that the cavity could be fully inserted into the spheroids during puncture. The hollow needles were incubated in 1 mg/mL DOX hydrochloride (Sigma-Aldrich) solution overnight for drug loading before use.

### Cell Culture, Biocompatibility Tests, and Cell Staining Experiments.

Human healthy fibroblast cells, CRL-2522, were obtained from the American Type Culture Collection (ATCC). The fibroblast cells were cultured in Minimum Essential Media (MEM, Gibco) supplemented with 10% (vol/vol) fetal bovine serum, penicillin (50 UI/mL), and streptomycin (50 μg/mL) in humidified, 37 °C, 5% CO_2_ 75 cm^2^ polystyrene cell culture flasks. For the staining experiments, the fibroblasts were seeded into µ-Slide four-well chambered Ibidi Polymer (ibiTreat) coverslips at the concentration of 10^5^ cells per 700 µL with the presence of the biodegradable needles. The viability of the cells was examined using LIVE/DEAD Viability/Cytotoxicity Kit (Invitrogen, Thermo Fisher Scientific), after 72 h with a fluorescence microscope (Nikon Eclipse Ti-E). For the confocal microscopy images, the cells were stained after 72 h of treatment. The actin filaments and nuclei of the cells were stained using ActinGreen 488 (Invitrogen, Thermo Scientific) and Hoechst 33342 (Thermo Scientific). The cells were fixed with 4% paraformaldehyde solution for 10 min and permeabilized with 0.1% Triton X-100 in PBS 1× for another 10 min. The cells then were incubated with ActinGreen 488 diluted in PBS 1× for 30 min and 1 µg/mL Hoechst 33342 dye diluted in PBS 1× for another 10 min. Then, the cells were analyzed by a confocal microscopy (Leica SP8 confocal microscope).

For the dose-dependent viability tests, the fibroblasts were seeded into a 96-well plate at the concentration of 10^4^ cells per well. The sterilized powders (made from the raw material of the biodegradable needles) dispersed in the cell culture media were added to the cells 24 h after seeding. After 72 h of adding the powder, the viability of the cells was analyzed by CellTiter-Glo 3D Cell Viability Assay (Promega), using 1:1 media to reagent ratio. The luminescence was measured in opaque 96-well plates with a plate reader (BioTek Gen5 Synergy 2). The relative percentage cell viability was calculated against the control/untreated group.

Epithelial colon tumor cells, HT-29, were obtained from the ATCC. The spheroids of HT-29 were fabricated using ultralow-attachment spheroid plates (Gibco), by seeding 2,000 cells per well. After 3 days of culture in low-attachment plates, the spheroids were transferred to cell culture dishes to facilitate attachment of spheroids to the substrate for the drug release experiments. After 2 days of transferring the spheroids into the cell culture dishes, the hollow needles with loaded drugs were punched into the HT-29 spheroids with a micromanipulator. The time lapse images of the spheroids were captured with an optical fluorescence microscope with an incubator (Zeiss Axio Observer A1, Carl Zeiss) for 8 h at 1-min intervals.

### Silk Needle Fabrication.

Silk fibroin (SF) was extracted from *Bombyx mori* cocoons, following a method reported in literature. First, a degumming process was done by adding 5 g of shredded cocoons into a glass beaker with 2 L boiled 0.02 M sodium carbonate/DI water solution and kept boiling for 30 min. This step was used to remove the sericin glue binding the SF fibers together. After degumming, the SF fibers were cleaned by soaking in 2 L DI water for 20 min. This step was repeated two more times. The rinsed SF fibers were dried overnight and then dissolved in 9.3 M lithium bromide (LiBr)/DI water solution with a concentration of 20 wt/vol%. For example, for 2 g of dried SF fiber, one needs to add 8 mL of 9.3 M LiBr solution. The SF/LiBr solution was kept in a 60 °C oven for a few hours until all the SF fibers totally dissolved and the color of the solution turned amber. This solution was then dialyzed in DI water for 48 h with eight changes of water to remove the salt to obtain SF aqueous solution. To remove the solid residues, the solution was centrifuged at 8,000 rpm for 20 min at 4 °C. This step was repeated until no visible impurities were left. The final SF solution had a concentration of ∼6 wt/wt%. To obtain a more concentrated SF solution, the solution was dialyzed again for 24 h but this time in a 10 wt/vol% PEG/DI water solution. The final SF solution had a concentration of 15 wt/wt%.

Silk needles were fabricated by molding. Needles were first printed on a stereolithography printer (SLA, Form 3, Formlabs) with the Clear Resin. Then, 10:1 PDMS was poured on the needles and then left to cure at 90°C  for 2 h. The negative PDMS molds were extracted and used without additional surface treatment. Then, 15 wt/wt% SF solution was poured on the molds. The bubbles trapped in the needle structure were removed by vacuuming, sonicating, and mechanically agitating the bubbles with a needle. After all the mold space was completely filled with SF solution, the mold was left on the bench overnight to dry. The dried needles could then be easily peeled off from the mold. At this point, the silk needles were water dissolvable due to the dominated random coil secondary conformation. To induce more mechanically robust and water-nondissolvable β-sheet, the silk needles were soaked in methanol for 24 h and then baked in a 90 °C oven for 12 h. The color of the needles turned from transparent to light yellow.

### Statistical Analysis.

All quantitative experimental values were presented as mean ± standard deviation (SD) of the mean.

## Supplementary Material

Supplementary File

Supplementary File

Supplementary File

Supplementary File

Supplementary File

## Data Availability

All study data required to reach the conclusions in this work are included in the article and/or supporting information.
