# Supplementary material for: On-demand anchoring of wireless soft miniature robots on soft surfaces
Source: Proc Natl Acad Sci U S A. 2022 Aug 15;119(34):e2207767119. doi: 10.1073/pnas.2207767119 (PMC9407667; doi:10.1073/pnas.2207767119)
Supplement: Supplementary File [file pnas.2207767119.sapp.pdf]

PNAS

Supplementary Information for

On-demand anchoring of wireless soft miniature robots on soft surfaces

Ren Hao Soon[1,2†], Ziyu Ren[1,2†], Wenqi Hu [1†], Ugur Bozuyuk[1,2], Erdost Yildiz[1], Meng Li[1] and Metin Sitti[1,2,3*]

[1] Physical Intelligence Department, Max Planck Institute for Intelligent Systems, 70569 Stuttgart, Germany

[2] Institute for Biomedical Engineering, ETH Zürich, 8092 Zürich, Switzerland

[3] School of Medicine and College of Engineering, Koç University, 34450 Istanbul, Turkey

[†] Equally contributing first authors

This PDF file includes:

Supplementary text
Figures S1 to S13
Tables S1 to S4
Legends for Movies S1 to S4
SI References

Other supplementary materials for this manuscript include the following:

Movies S1 to S4

Supplementary Information Text

1. Theoretical force provided by a magnetic soft robot

With a remanence of 85 emu/g (experimentally tested), Assuming robot with dimensions $3\ mm \times 1\ mm \times 0.2\ mm$,

$$V_{robot} = 3 \times 1 \times 0.2 \times 10^{-9}$$

$$= 0.6 \times 10^{-9}\ m^3$$

$$V_{Ecoflex} + V_{NdFeB} = 0.6 \times 10^{-9}\ m^3$$

$$\left(\frac{m}{\rho}\right)_{Ecoflex} + \left(\frac{m}{\rho}\right)_{NdFeB} = 0.6 \times 10^{-9}\ m^3$$

Assuming 1:3 mass ratio of Ecoflex to NdFeB,

$$m_{NdFeB}\left[\left(\frac{1}{3\rho}\right)_{Ecoflex} + \left(\frac{1}{\rho}\right)_{NdFeB}\right] = 0.6 \times 10^{-9}\ kg$$

$$\rho_{Ecoflex} = 1070\ kg/m^3\ ; \rho_{NdFeB} = 7610\ kg/m^3$$

$$m_{NdFeB} = 1.3546 \times 10^{-6}\ kg$$

$$Magnetic\ moment\ m = 85(1.3546 \times 10^{-6}) \times 10^3$$

$$= 1.1514 \times 10^{-1}\ emu$$

$$= 1.1514 \times 10^{-4}\ Am^2$$

Assuming an external field of 50 mT applied perpendicularly to magnetization profile of robot (uni-direction),

$$\tau = 50 \times 10^{-3} \times 1.1514 \times 10^{-4} \times \sin 90\ Nm$$

$$= 5.757 \times 10^{-6}\ Nm$$

Assuming a rigid body,

$$\tau = F.d$$

$$F = \frac{\tau}{d} = \frac{5.757 \times 10^{-6}}{3 \times 10^{-3}} = 1.919 \times 10^{-3} = 2\ mN$$

2. Analysis of the mechanism

Based on high-speed images of the trigger event (Fig. S3A), the puncturing process was divided into six phases: initial, deflection, puncture, needle insertion, recoil and final phase (Fig. S3B). We modelled the mechanism as a spring-mass system, with the soft surface and spring being modelled as serial springs (Fig. S3C). As the time taken for anchoring is less than a second (Fig. 1E), the response of the surface is dominantly elastic and can be characterized by a linear stress-strain response (i.e., spring) (*1*). In the initial phase (phase I), the mechanism is oriented towards the surface and there are no net forces acting on the soft surface or the needle. We set the origin to be at the soft surface and define x_1 as the maximum surface deflection. x_1 need not always be at the needle tip as seen in the illustrations of the later stages (phases IV and V). In the deflection phase (phase II), the mechanism is triggered and the spring drives the needle into the surface by x_1. There is no cutting or penetration of the surface in this phase. When x_1 reaches a value such that the corresponding stress intensity factor that accompanies the stress field induced at the needle tip exceeds the fracture toughness of the surface, the needle penetrates the surface (phase III). The x displacement at this point is recorded as x_p. Frictional force will start to act from this phase onwards. Since friction acts along the length of the needle, we assume that the pressure acting along the length of the needle is constant and is proportional to the depth of insertion $x_1 - x_p$. This process continues until the copper plate driving the needle impacts the surface or until the needle stops if the needle is sufficiently long. At this point (phase IV), deflection of the surface is maximum and the velocity of m_1, which corresponds to the mass of the copper plate, is zero. After this point, the spring loses contact with m_1 and the surface starts releasing the stored elastic energy. This accelerates m_1 in the opposite direction until all the energy is released (phase V). In all these phases, since the casing is not fixed and is free to move, the casing accelerates in the opposite direction.

By examining the equations of motion of each phase (next section), a system of ordinary differential equations describing the motion of the head and the casing during the process was obtained. Equations corresponding to the deflection phase (phase I to phase II) were then numerically solved

in MATLAB using the ode45 solver. The stiffness of the soft surfaces were computed from the experimentally determined Young's modulus based on a previously proposed method (*2*). These surfaces were selected to cover the stiffness of living tissues in the kPa range, which encompasses most organs inside the human body (*3*). For the spring constants, we directly used the values provided by the manufacturer for the springs. To increase the accuracy of the model, parameters used in the simulations were experimentally determined. All other simulation parameters are provided in Table S2. In the simulations, we only considered the case where a barbless needle, placed right next to the surface, enters the surface at 90°. When the angle of insertion or distance from the surface changes, this results in a reduction in the force supplied to deform the substrate. Similarly, the effect of the barb can be reflected in this model by introducing a penalty term. Specifically, addition of a barb will increase the apparent bevel angle of the needle and reduce the force applied (*2*). As these affect all configurations equally and are not specific to a particular configuration, the same trends will hold and we do not consider their effects in the model.

3. Equations of motion for the various stages

Between 1 and 2:

1: Initial state – full compression of spring, no deflection of substrate

2: Initial deformation – no cutting, pure deflection of substrate

$$Governing\ equation\ for\ this\ stage$$

$$\sum F_x = ma$$

$$\begin{bmatrix} m_1\ddot{x_1} \\ m_2\ddot{x_2} \end{bmatrix} = \begin{bmatrix} -k_{sub}x_1 + k_{sp}[L_{sp} - (x_1 - x_2) - D] \\ -k_{sp}[L_{sp} - (x_1 - x_2) - D] \end{bmatrix}$$

$$\begin{bmatrix} \ddot{x_1} \\ \ddot{x_2} \end{bmatrix} = \begin{bmatrix} -\frac{k_{sub} + k_{sp}}{m_1} & \frac{k_{sp}}{m_1} \\ \frac{k_{sp}}{m_2} & -\frac{k_{sp}}{m_2} \end{bmatrix} \begin{bmatrix} x_1 \\ x_2 \end{bmatrix} + \begin{bmatrix} \frac{k_{sp}}{m_1}(L_{sp} - D) \\ -\frac{k_{sp}}{m_2}(L_{sp} - D) \end{bmatrix}$$

$$where\ L_{sp}\ denotes\ the\ uncompressed\ length\ of\ spring,$$

$$D\ denotes\ the\ distance\ between\ the\ needle\ tip\ and\ the\ substrate$$

Between 2 and 3:

2: Initial deformation – no cutting, pure deflection of substrate

3: Needle insertion – combined cutting and spreading force (C), linear friction, substrate exerts constant pressure P on the needle, needle length L

$$Governing\ equation\ for\ this\ stage$$

$$\sum F_x = ma$$

$$\begin{bmatrix} m_1\ddot{x_1} \\ m_2\ddot{x_2} \end{bmatrix} = \begin{bmatrix} -k_{sub}x_1 + k_{sp}\left[L_{sp} - (x_1 - x_2) - D\right] - \mu_k N - C \\ -k_{sp}[L_{sp} - (x_1 - x_2) - D] \end{bmatrix}$$

$$\begin{bmatrix} m_1\ddot{x_1} \\ m_2\ddot{x_2} \end{bmatrix} = \begin{bmatrix} -k_{sub}x_1 + k_{sp}\left[L_{sp} - (x_1 - x_2) - D\right] - \mu_k P\pi d\left(x_1 - x_p\right) - C \\ -k_{sp}[L_{sp} - (x_1 - x_2) - D] \end{bmatrix}$$

$$\begin{bmatrix}\ddot{x_1}\\ \ddot{x_2}\end{bmatrix} = \begin{bmatrix}-\frac{k_{sub}+k_{sp}+\mu_k P\pi d}{m_1} & \frac{k_{sp}}{m_1}\\ \frac{k_{sp}}{m_2} & -\frac{k_{sp}}{m_2}\end{bmatrix}\begin{bmatrix}x_1\\ x_2\end{bmatrix} + \begin{bmatrix}\frac{k_{sp}}{m_1}\left(L_{sp}-D\right)+\frac{\mu_k P\pi d x_p}{m_1}-\frac{C}{m_1}\\ -\frac{k_{sp}}{m_2}(L_{sp}-D)\end{bmatrix}$$

$$where\ x_{1\,puncture}\ denotes\ the\ displacement\ of\ the\ needle\ at\ puncture,$$

$$N\ denotes\ the\ normal\ contact\ force\ along\ the\ length\ of\ the\ needle$$

At 4:

4: Maximum deflection – no net force on m_1, copper plate touches substrate

$$\sum F_x = 0$$

$$\begin{bmatrix}0\\ m_2\ddot{x_2}\end{bmatrix} = \begin{bmatrix}-k_{sub}x_1 + k_{sp}\left[L_{sp}-(x_1-x_2)-D\right]-\mu_k N - N_{copper}\\ -k_{sp}[L_{sp}-(x_1-x_2)-D]\end{bmatrix}$$

$$where\ N_{copper}\ denotes\ the\ normal\ contact\ force\ on\ the\ copper\ plate$$

After 4:

4: Release of energy from substrate – no cutting, pure deflection of substrate, spring loses contact with m_1

$$Governing\ equation\ for\ this\ stage$$

$$\sum F_x = ma$$

$$m_1\ddot{x_1} = -k_{sub}x_1 + \mu_k N$$

4. Initial biocompatibility studies

To demonstrate the biocompatibility of long-term anchoring on tissue surfaces, we cultured the needles with a human fibroblast cell line. We seeded the fibroblast cells on top of the needles, and observed their viability after 72 hours of culture. The fibroblasts demonstrated clear viability in the culture environment and at the interface of the needle while having spindle-shaped, healthy morphology (Fig. S11A). The confocal microscopy analysis also revealed the 3D migration of fibroblasts towards the needle surface after 72 hours, demonstrating the biocompatibility of the needle interface (Fig. S11B, C). To demonstrate that the needles and the by-products released during degradation were non-toxic (Fig. S11D), the base material of the needle was grounded into powder and cultured with the fibroblasts at different concentrations. The cell viability analysis showed that the by-products of degradation (up to the concentration of 600 $\mu g/mL$) did not cause any adverse effect on the cells after 72 hours, demonstrating the versatility and compatibility of the material (Fig. S11E).

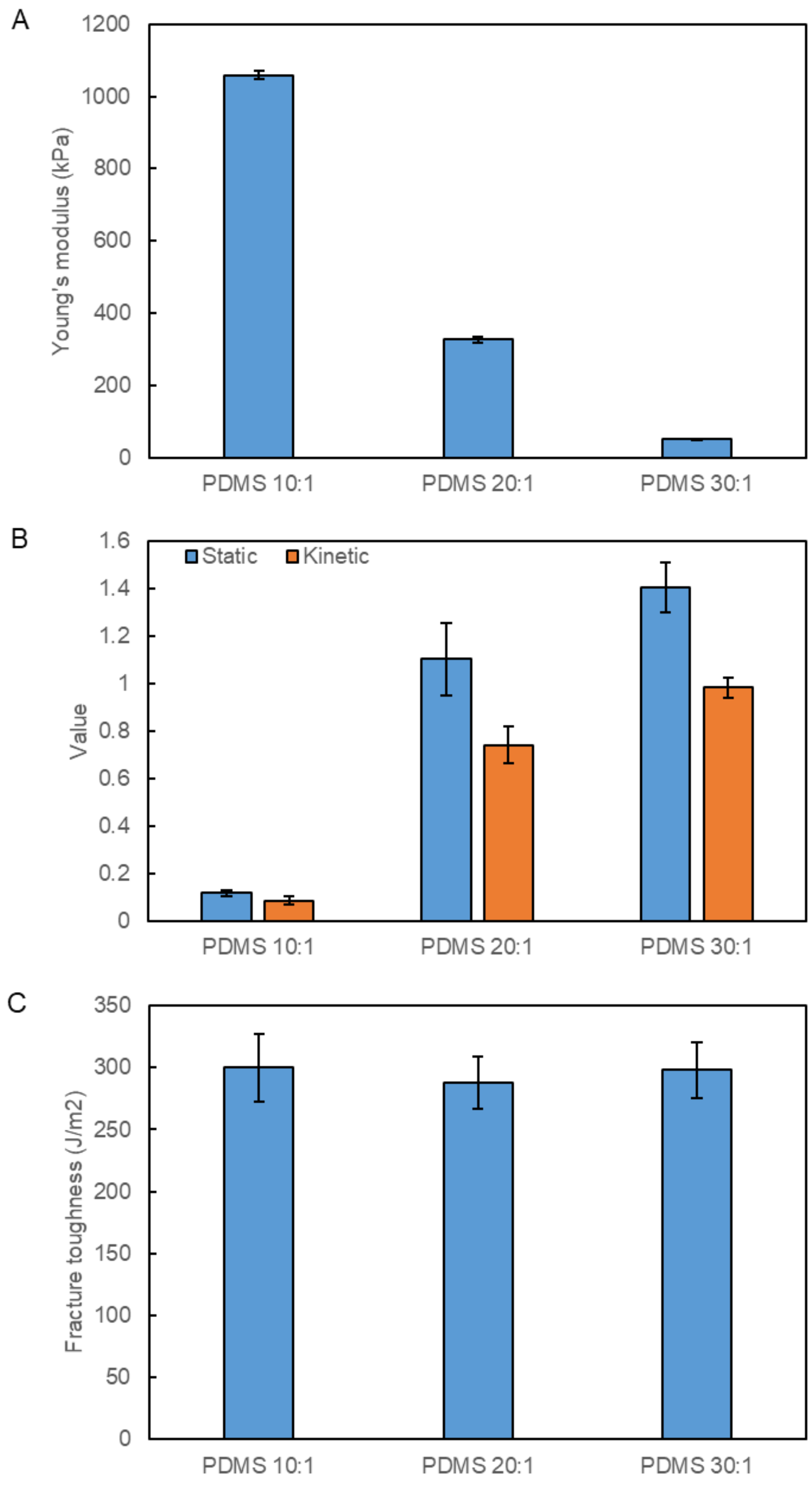

Figure S1. Material properties of the substrates used in this work (n=3). (**A**) Young's modulus. (**B**) Coefficient of static and kinetic friction. (**C**) Fracture toughness. Error bars represent the standard deviation.

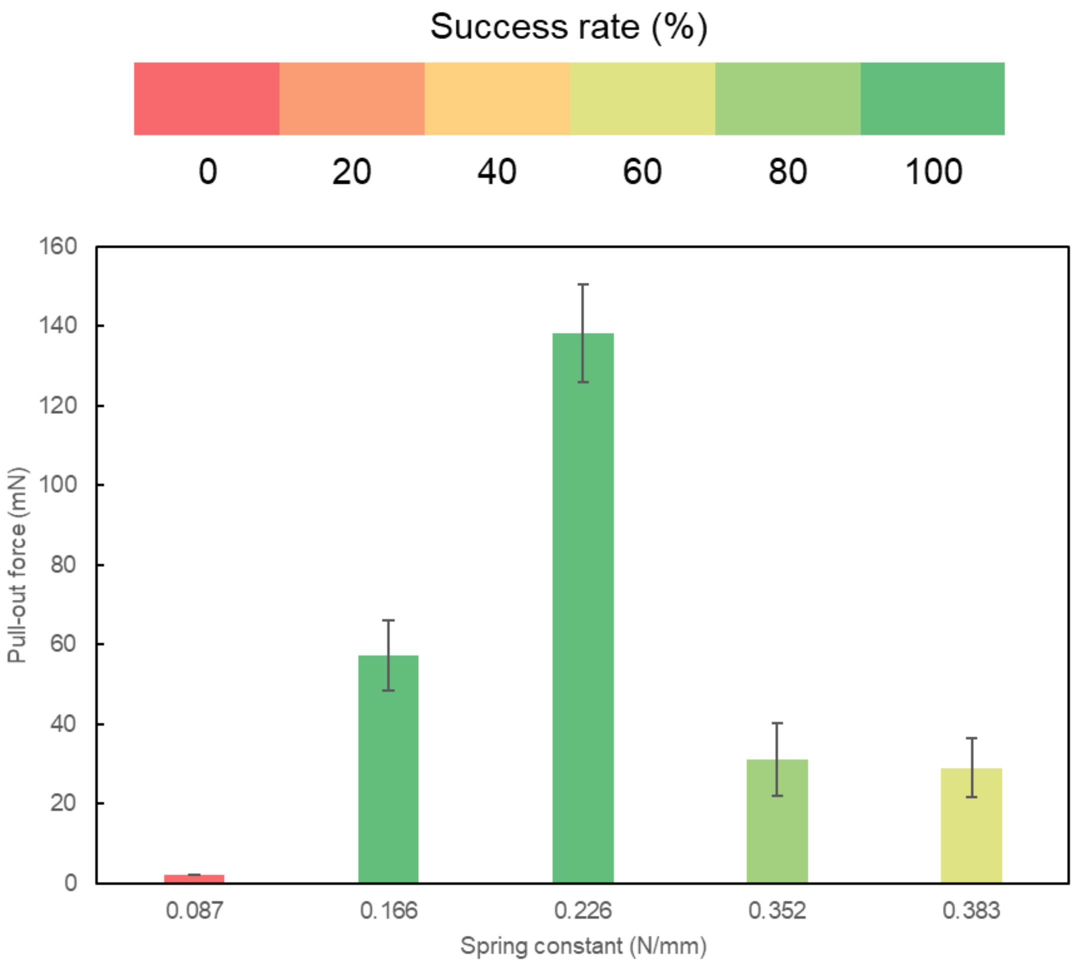

Figure S2. Graph of measured pull-out forces against spring constants (n=5). Error bars represent the standard deviation.

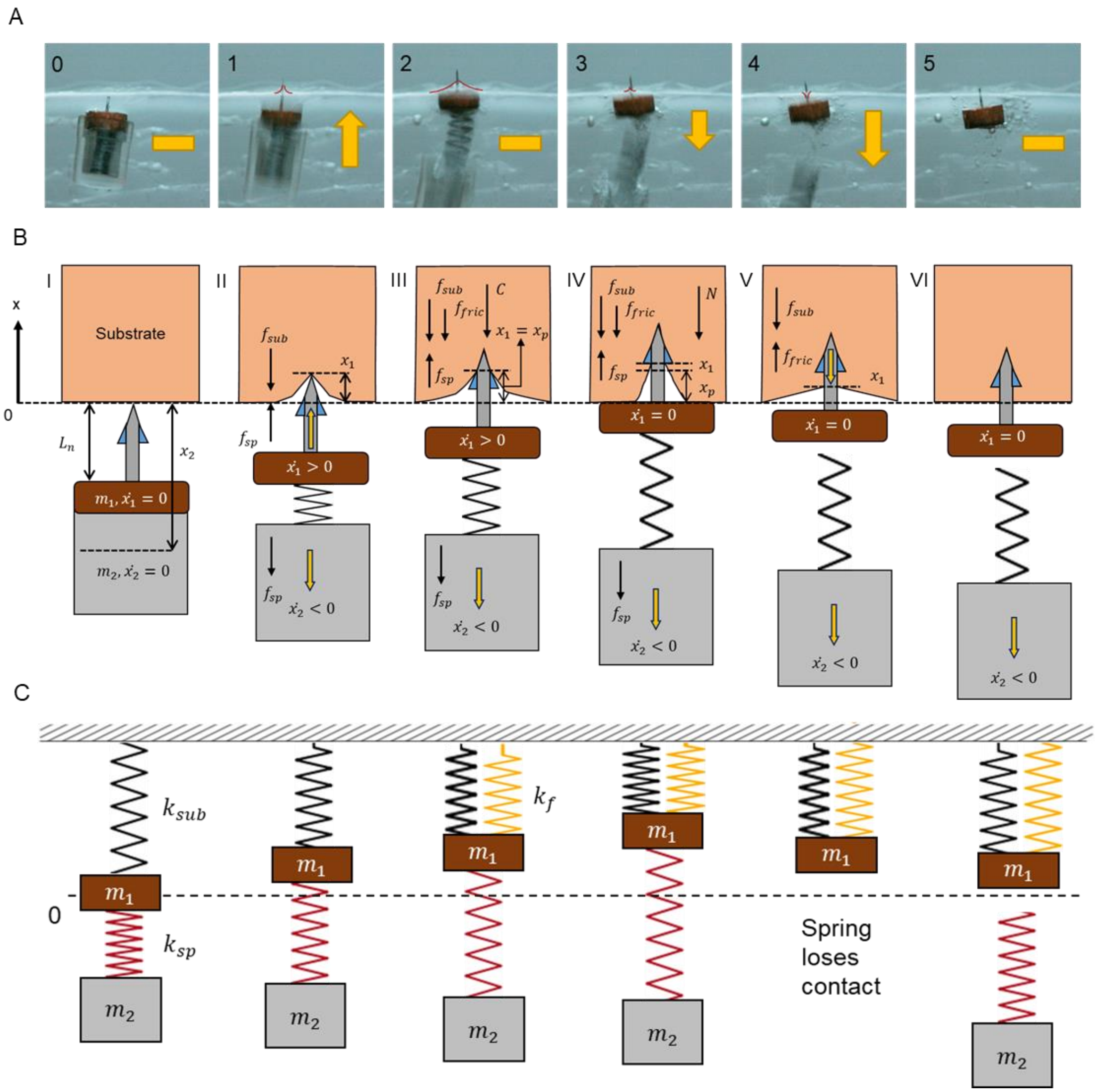

Figure S3. System-based model of the anchoring mechanism for optimization. (**A**) Actuation sequence of the mechanism into 30:1 PDMS. Yellow arrow indicates direction of motion. Red markings indicate deflection of the substrate. (**B**) Illustration depicting the six stages of the tissue penetration. (**I**) Positioning of the mechanism. (**II**) Initial substrate deflection. (**III**) Tissue puncture. Friction starts acting from this stage. (**IV**) Maximum penetration into substrate. Spring loses contact with the head after this stage. (**V**) Elastic energy release of substrate. (**VI**) Anchoring of copper plate to substrate. Yellow arrows depict direction of motion. Black arrows indicate the forces acting on the respective bodies. (**C**) Equivalent spring mass system of the process.

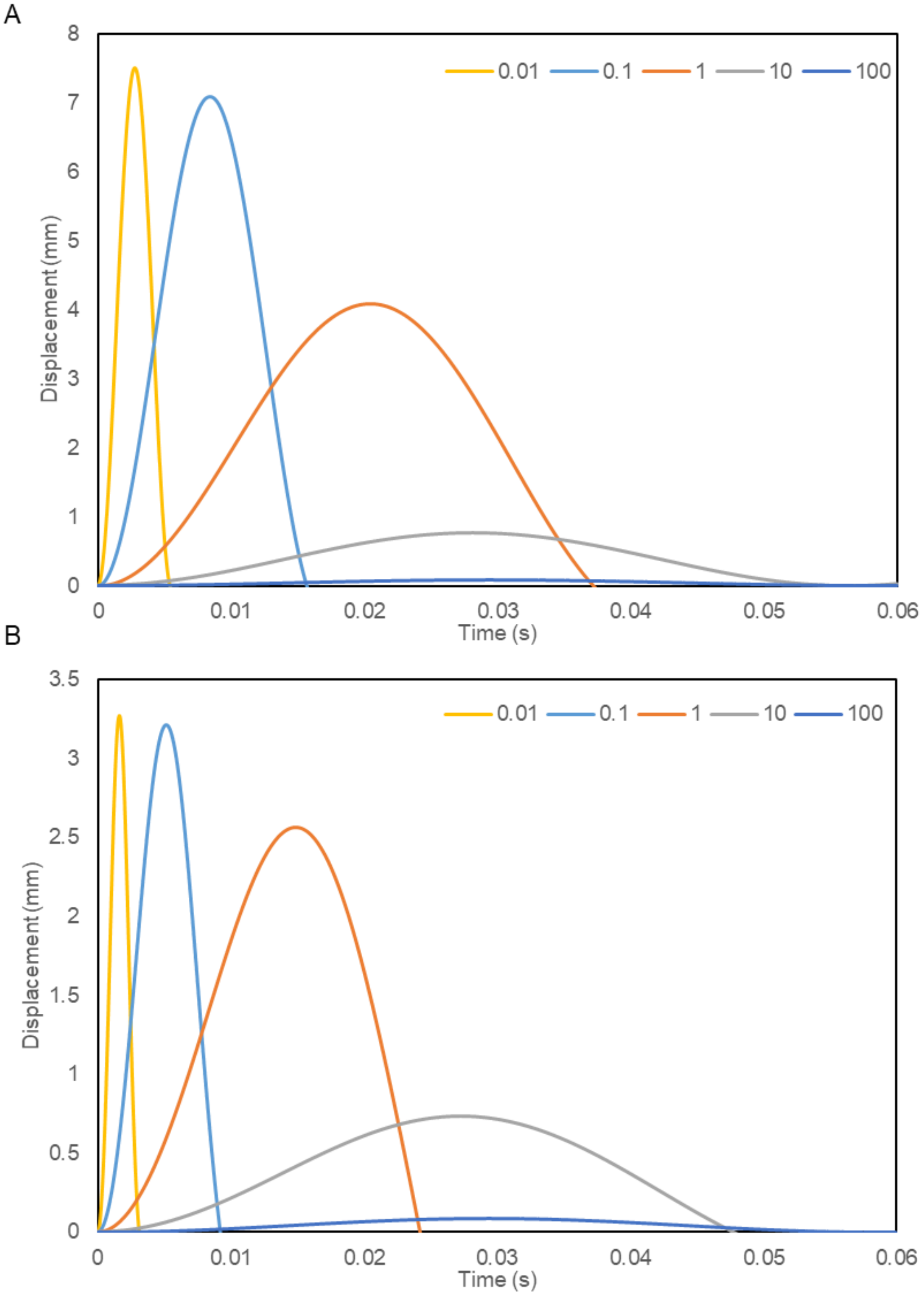

Figure S4. Displacement-time graph of the mass m_1 at different $\frac{m_1}{m_2}$ mass ratios travelling into the substrate during phase I. The colors represent the different mass ratios. (**A**) 30:1 PDMS. (**B**) 10:1 PDMS.

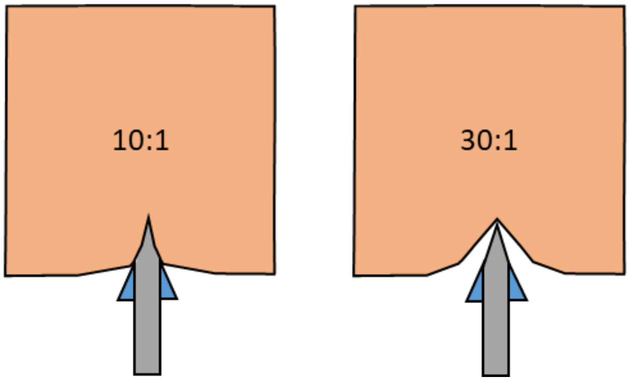

Figure S5. Illustrated deformation of the soft substrate when an identical mechanism is used. 10:1 PDMS (left) and 30:1 PDMS (right).

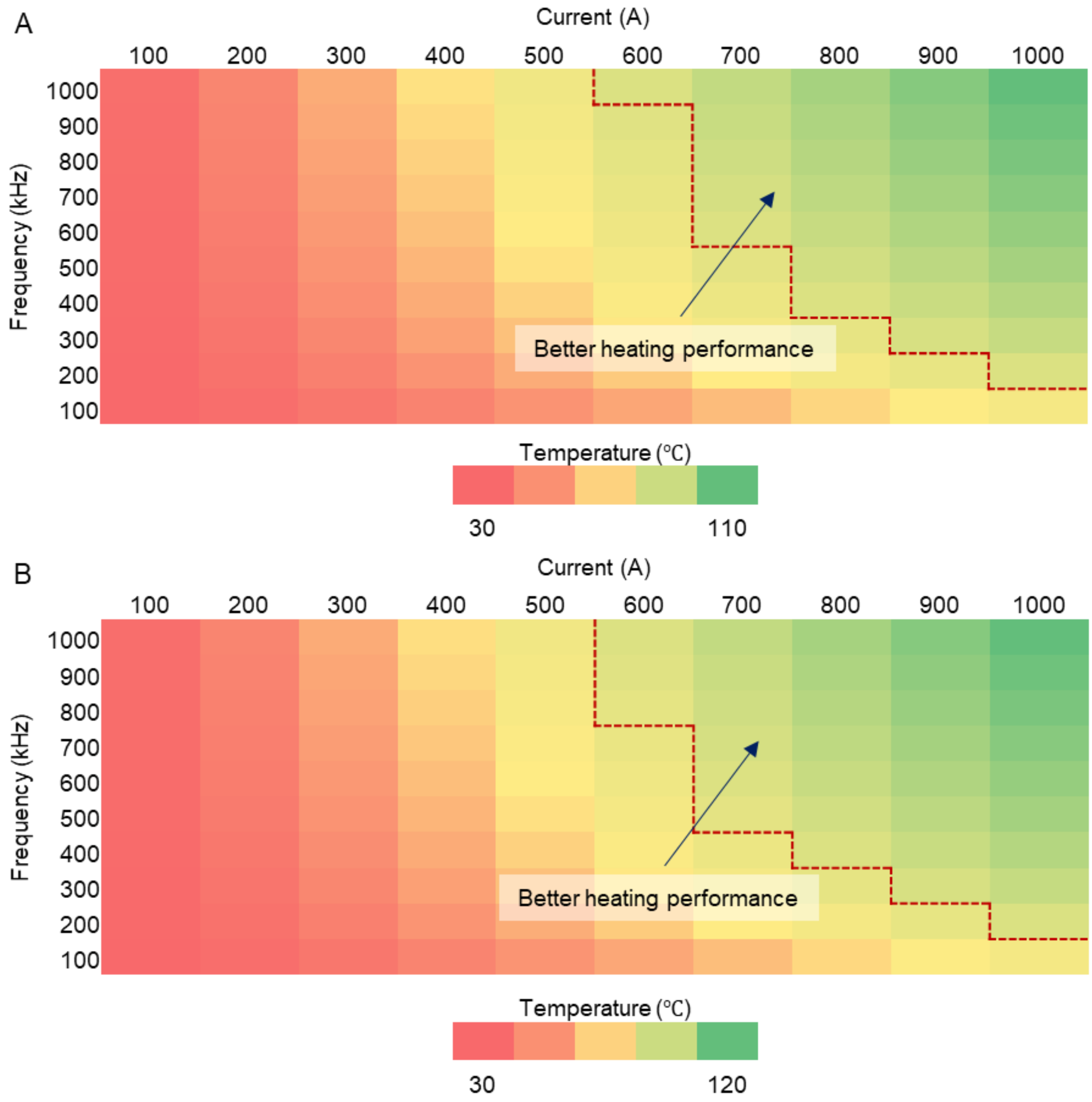

Figure S6. Effect of the RF frequency and input current amplitude on the induced temperature of the copper plate. (**A**) Predicted temperature of the copper plate after 25 s at a constant angle ϕ = 0° and distance of 0.03 m. Red dotted line indicates that the temperature has exceeded 60℃. (**B**) Predicted temperature of the copper plate after 25 s at a constant angle ϕ = 90° and distance of 0.03 m. Red dotted line indicates that the temperature has exceeded 60℃.

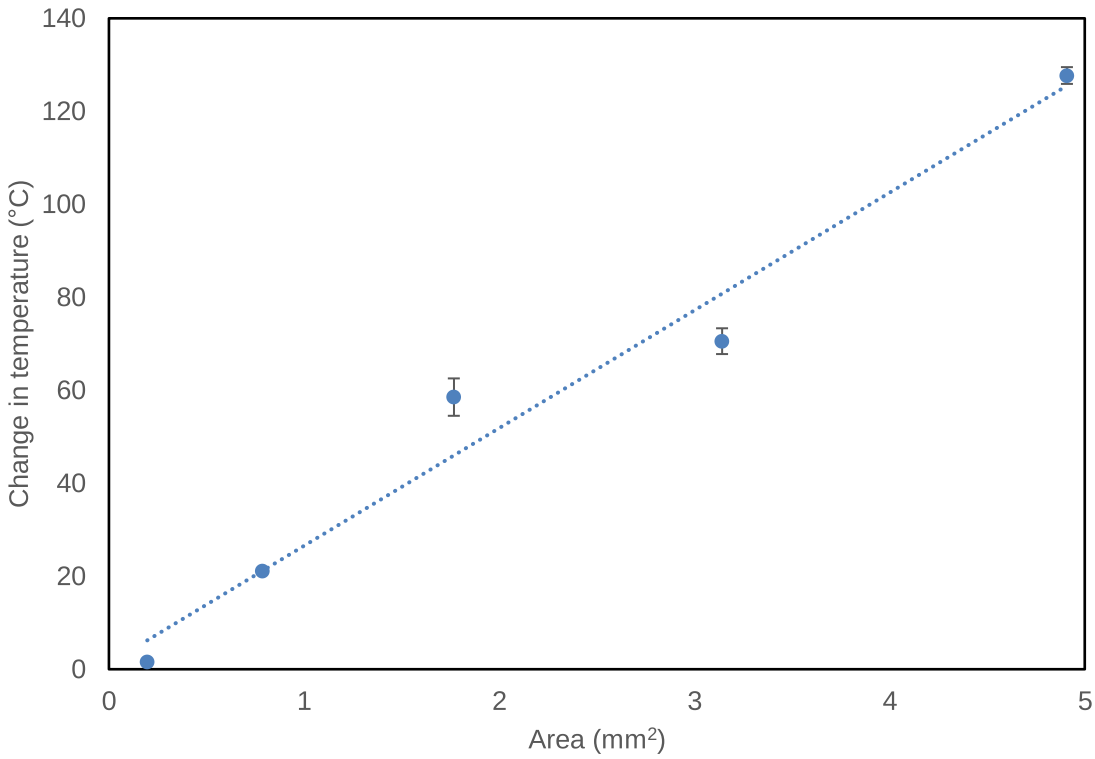

Figure S7. Graph of temperature against diameter of the copper plate after 25 s of RF exposure at a distance of 0.02 m (n=3). Error bars represent the standard deviation.

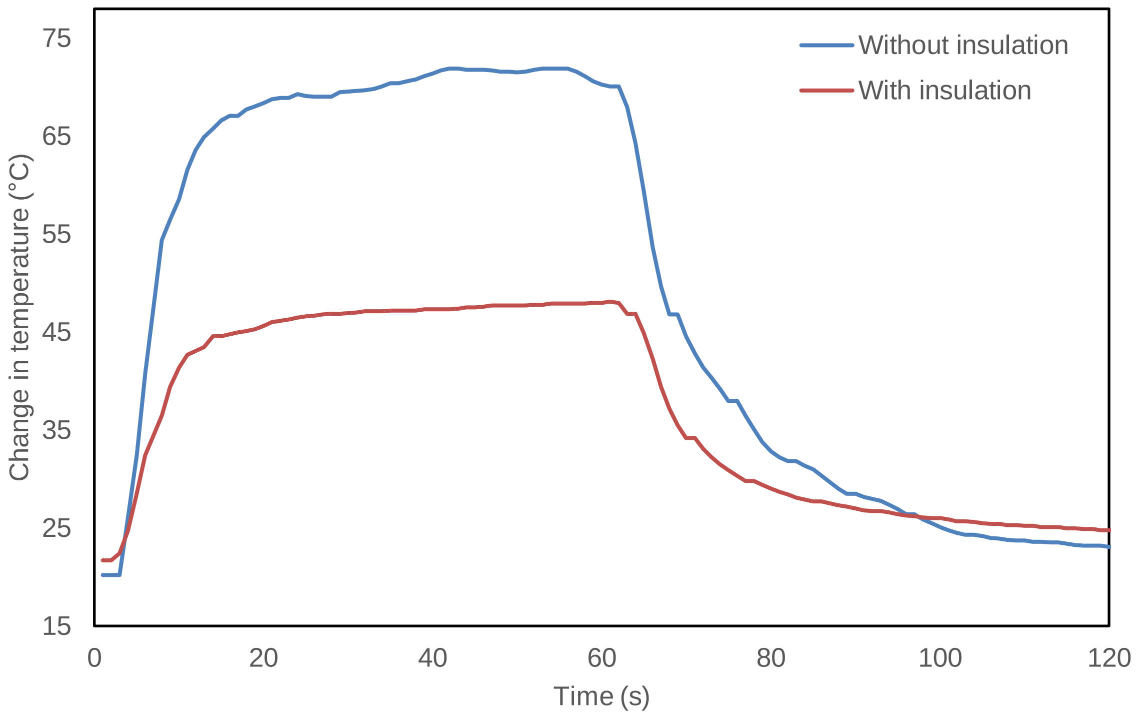

Figure S8. Temperature against time graph of the outer surface of the copper plate with and without insulation. Device is submerged in DI water.

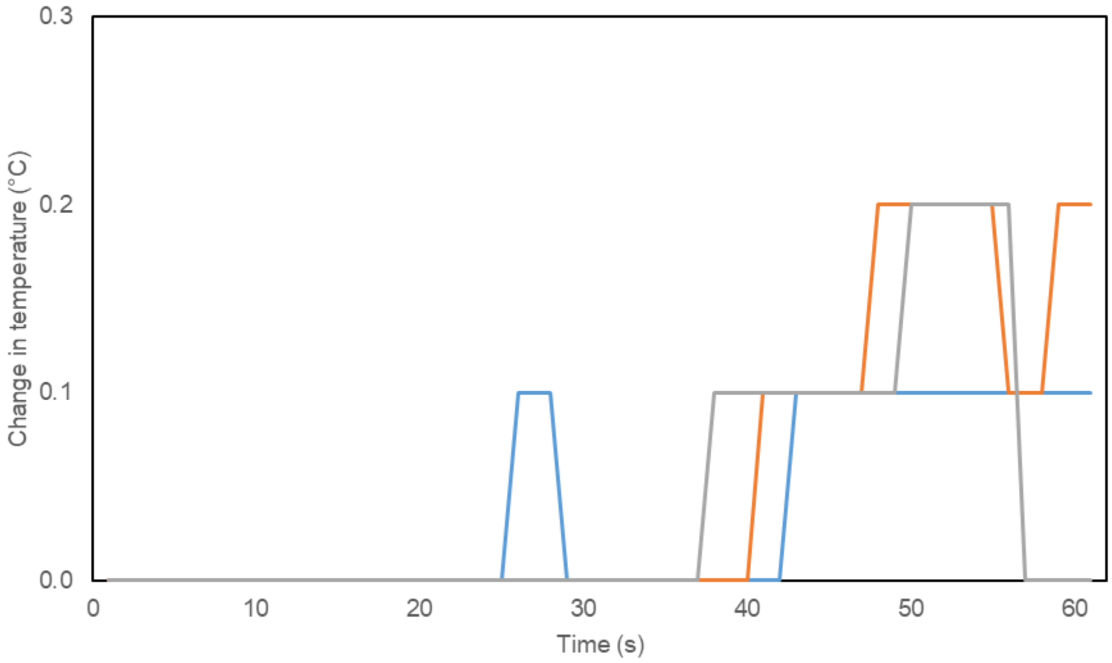

Figure S9. Temperature against time graph of porcine bladder tissue when subjected to the RF field for a minute (n=3).

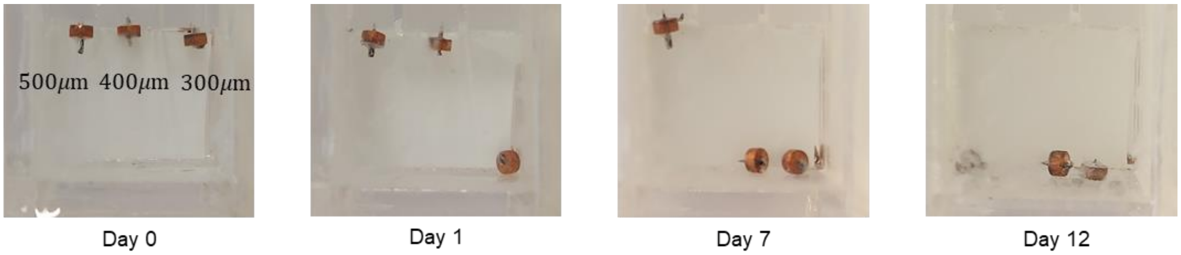

Figure S10. Biodegradable needles anchored on a PDMS 10:1 substrate and submerged in water at 37 ℃. (Left to right) Image on day 0 was taken immediately after anchoring.

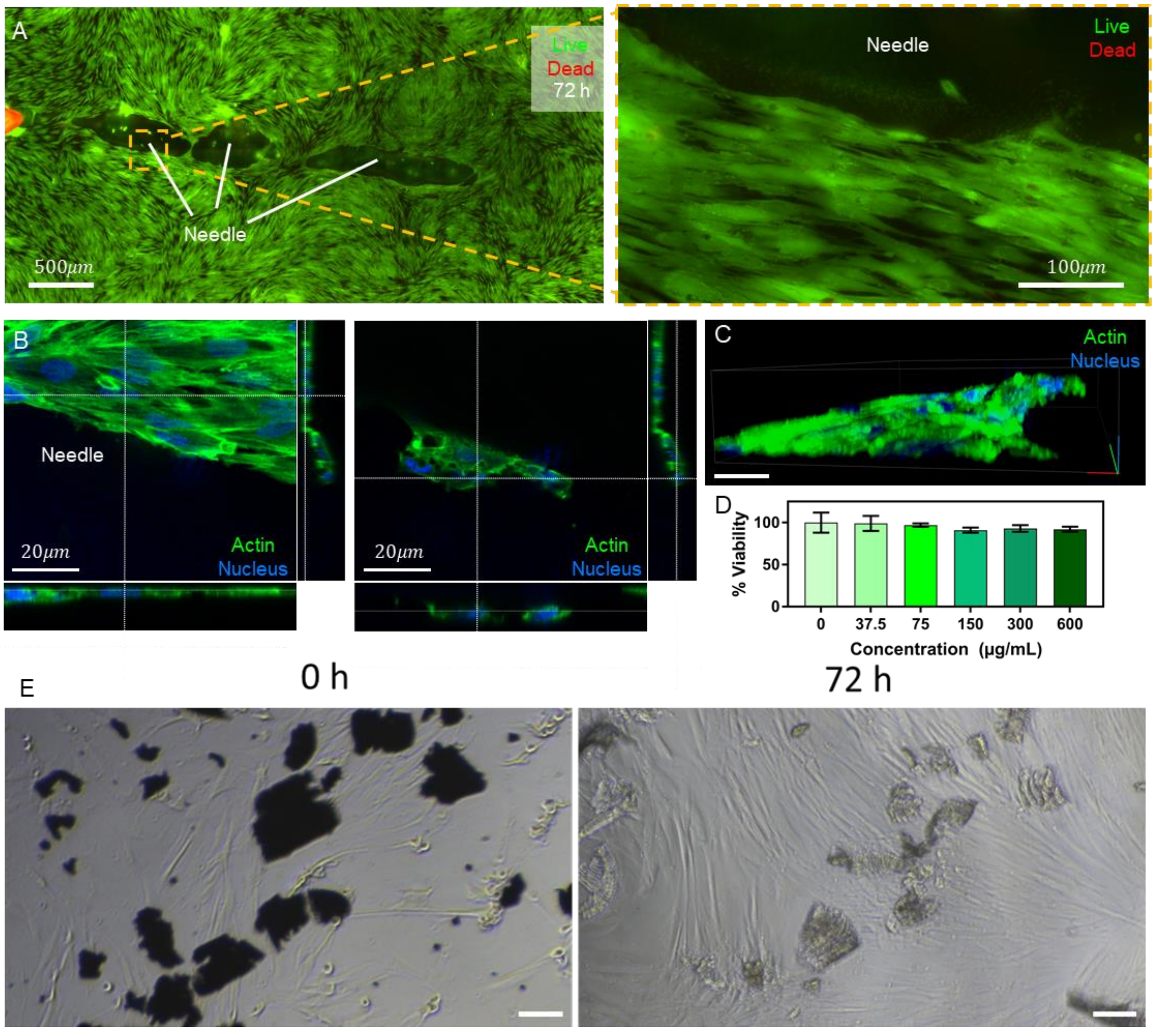

Figure S11. Results from the initial biocompatibility tests. (**A**) Live-dead staining of fibroblast cells cultured with the biodegradable needle. The cells were fully viable after 72 hours of culture and displayed healthy morphology even at the interface of the needle. (**B**) Confocal microscopy analysis of the cells at the interface of the needle. The left and right images show the different z-levels of the same image, the latter being higher level. The right image shows grown cells towards +z direction on the needle. (**C**) 3D constructed confocal microscopy image showing the cell growth on +z direction towards to the needle. (**D**) Cell viability as a function of the needle powder concentration after 72 hours of treatment, based on adenosine triphosphate (ATP) production. The cells showed more than 90% viability even at relatively high concentrations. Error bars represent the standard deviation. (**E**) Degradation of the needle (powdered) after 72 hours. The images were taken from random areas of the wells. The shape and the color of the powder particles have dramatically changed due to degradation. Scale bars: 200 μm.

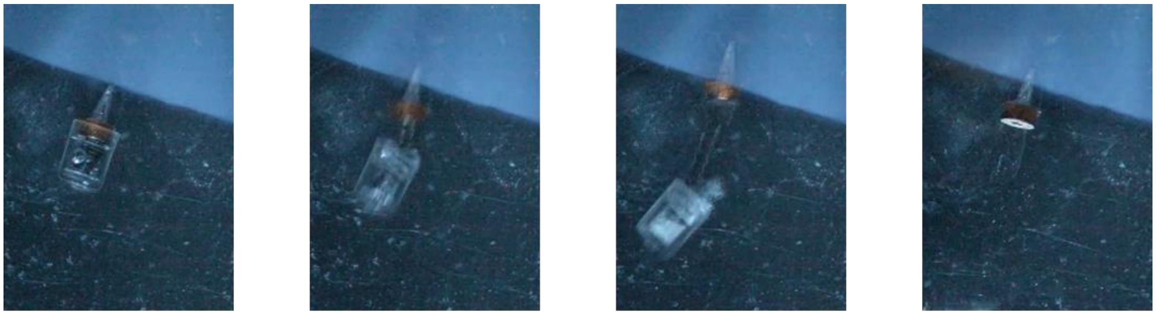

Figure S12. Actuation sequence of a silk needle puncturing an agarose substrate (2% by weight). t refers to the time after actuation and not the duration of RF field applied.

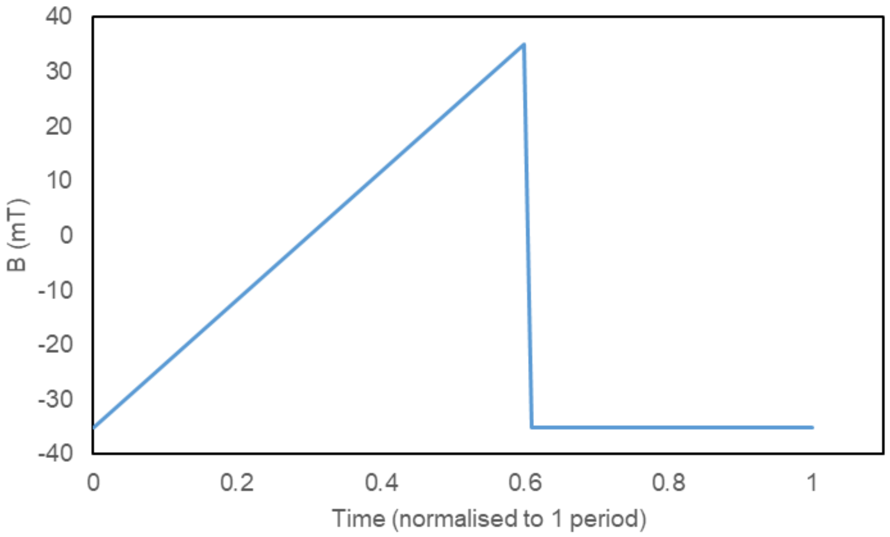

Figure S13. Magnetic field waveform used to actuate the jellyfish soft robot.

Table S1. Comparison of the forces required for tissue penetration across literature. Note that these values were obtained indirectly (i.e. based on the forces provided by the respective devices) and may not necessarily reflect the actual forces required for tissue penetration.

Mechanism	Needle specifications	Maximum penetration depth/mm	Penetration force/N	Tissue type	Reference
Spring-loaded	Single needle – cone angle 19.4°	15 mm	O(1)	Stomach (porcine)	(*4*)
Spring-loaded	96 conical microneedles – cone angle 11.3°	1 mm	O(0.1)	Small intestine (porcine)	(*5*)
MRI powered Gauss Gun	18, 20, 26 G needles	15 mm	O(1)	Agarose gel	(*6*)
Magnetic gradient	24 G needle	10 mm	0.4-0.6 N	Stomach (porcine)	(*7*)
Magnetic hammer	Single needle – cone angle 11.3°	NA	Pulsing force O(0.01)	Brain (goat)	(*8*)
This work	Barbed single needle – cone angle 10°	1 mm	O(1)	Bladder (porcine)	-

Table S2. Simulation parameters.

Parameter	Value (unit)
m_1	20 mg
m_2	30 mg
L_{sp}	6 mm
x_1 at t=0	0 mm
$\dot{x_1}$ at t=0	0 m/s
x_2 at t=0	-1.8 mm
$\dot{x_2}$ at t=0	0 m/s
$\mu_k\ - casing\ on\ glass$	0.1609
C_d	1.15
ρ_{water}	1000 kg/m^3

Table S3. Comparison of the pull-out forces of barbed needles in biological tissues across literature.

Design	Height/ mm	Diameter/ um	Penetration force/ N	Pull-out force/ N	Reference
Honeybee	2	200	0.125 (rabbit skin)	0.073 (rabbit skin)	(*9*)
Backwards barbs	4	400	0.416 (chicken)	0.176 (chicken)	(*10*)
Porcupine	5	200	0.03 (muscle)	0.031 (pig skin)	(*11*)
Swelling tip	0.7	280	0.092 (pig skin)	0.012 (pig skin)	(*12*)
This work	1	100	0.95 (regardless of substrate)	0.036 (pig stomach) 0.025 (pig bladder)	-

Table S4. Materials used for the mechanism in this work and their biocompatible equivalents.

Part	Current material	Biocompatible equivalent	Remarks
Casing	Clear Resin (Formlabs)	BioMed Clear Resin (Formlabs)	USP Class VI certified
Spring	Elgiloy	-	Used in implants, orthodontic wires, medical instruments, orthopedic fixtures
Copper plate	Copper	-	Used in intrauterine devices
Needle	Stainless steel	Mg-based alloy/ Silk	Stainless steel is used in implants.<br>The Mg-based alloy/ silk is biocompatible and biodegradable.
Barbs	IP-S (Nanoscribe GmbH)	IP-Visio (Nanoscribe GmbH)	ISO 10993-5 / USP 87 non-cytotoxic certified
Cyanoacrylate adhesive	Loctite 401/431 (Loctite)	Dermabond (J&J)	Used for wound closures

Movie S1 (separate file). Cyclic loading of bladder – 100 cycles @ 100% strain

Movie S2 (separate file). Integration with jellyfish robot – 35 mT @ 35 Hz

Movie S3 (separate file). Ultrasound guided device

Movie S4 (separate file). Drug delivery into tumor spheroid

References

1. O. Thoumine, A. Ott, Time scale dependent viscoelastic and contractile regimes in fibroblasts probed by microplate manipulation. *J. Cell Sci.* **110**, 2109–2116 (1997).
2. S. Jiang, P. Li, Y. Yu, J. Liu, Z. Yang, Experimental study of needle–tissue interaction forces: Effect of needle geometries, insertion methods and tissue characteristics. *J. Biomech.* **47**, 3344–3353 (2014).
3. C. F. Guimarães, L. Gasperini, A. P. Marques, R. L. Reis, The stiffness of living tissues and its implications for tissue engineering. *Nat. Rev. Mater.* **5**, 351–370 (2020).
4. A. Abramson, E. Caffarel-Salvador, M. Khang, D. Dellal, D. Silverstein, Y. Gao, M. R. Frederiksen, A. Vegge, F. Hubálek, J. J. Water, A. V. Friderichsen, J. Fels, R. K. Kirk, C. Cleveland, J. Collins, S. Tamang, A. Hayward, T. Landh, S. T. Buckley, N. Roxhed, U. Rahbek, R. Langer, G. Traverso, An ingestible self-orienting system for oral delivery of macromolecules. *Science.* **363**, 611–615 (2019).
5. A. Abramson, E. Caffarel-Salvador, V. Soares, D. Minahan, R. Y. Tian, X. Lu, D. Dellal, Y. Gao, S. Kim, J. Wainer, J. Collins, S. Tamang, A. Hayward, T. Yoshitake, H.-C. Lee, J. Fujimoto, J. Fels, M. R. Frederiksen, U. Rahbek, N. Roxhed, R. Langer, G. Traverso, A luminal unfolding microneedle injector for oral delivery of macromolecules. *Nat. Med.* **25**, 1512–1518 (2019).
6. A. T. Becker, O. Felfoul, P. E. Dupont, in *2015 IEEE International Conference on Robotics and Automation (ICRA)* (IEEE, 2015; http://ieeexplore.ieee.org/document/7139341/), vols. 2015-June, pp. 1184–1189.
7. D. Son, H. Gilbert, M. Sitti, Magnetically Actuated Soft Capsule Endoscope for Fine-Needle Biopsy. *Soft Robot.* **7**, 10–21 (2020).
8. J. Leclerc, A. Ramakrishnan, N. Tsekos, A. Becker, Magnetic Hammer Actuation for Tissue Penetration using a Millirobot. *IEEE Robot. Autom. Lett.* **3**, 1–1 (2017).
9. Z. Chen, Y. Lin, W. Lee, L. Ren, B. Liu, L. Liang, Z. Wang, L. Jiang, Additive Manufacturing of Honeybee-Inspired Microneedle for Easy Skin Insertion and Difficult Removal. *ACS Appl. Mater. Interfaces*. **10**, 29338–29346 (2018).
10. D. Han, R. S. Morde, S. Mariani, A. A. La Mattina, E. Vignali, C. Yang, G. Barillaro, H. Lee, 4D Printing of a Bioinspired Microneedle Array with Backward- Facing Barbs for Enhanced Tissue Adhesion. *Adv. Funct. Mater.* **30**, 1909197 (2020).
11. W. K. Cho, J. A. Ankrum, D. Guo, S. A. Chester, S. Y. Yang, A. Kashyap, G. A. Campbell, R. J. Wood, R. K. Rijal, R. Karnik, R. Langer, J. M. Karp, Microstructured barbs on the North American porcupine quill enable easy tissue penetration and difficult removal. *Proc. Natl. Acad. Sci.* **109**, 21289–21294 (2012).
12. S. Y. Yang, E. D. O'Cearbhaill, G. C. Sisk, K. M. Park, W. K. Cho, M. Villiger, B. E. Bouma, B. Pomahac, J. M. Karp, A bio-inspired swellable microneedle adhesive for mechanical interlocking with tissue. *Nat. Commun.* **4**, 1702 (2013).
